# The Photoperiod Regulates Granulosa Cell Apoptosis through the FSH-Nodal/ALK7 Signaling Pathway in *Phodopus sungorus*

**DOI:** 10.3390/ani12243570

**Published:** 2022-12-16

**Authors:** Yan Qi, Hui-Liang Xue, Yun-Jiao Zheng, Yun-Fei Yin, Wen-Lei Xu, Jin-Hui Xu, Ming Wu, Lei Chen, Lai-Xiang Xu

**Affiliations:** School of Life Sciences, Qufu Normal University, Qufu 273165, China

**Keywords:** Nodal/ALK7 signaling pathway, FSH, granulosa cell, apoptosis, photoperiods, ovarian function

## Abstract

**Simple Summary:**

The photoperiod regulates the follicle development in the seasonal reproduction of animals through the HPO axis, which enables the offspring to be born in specific seasons to better adapt to the external environment. However, the specific mechanism remains unclear. Granulosa cells in the ovary are crucial for the follicle development and the maintenance of the ovarian function. This study focused on the structure of ovaries and the development state of the granulosa cells under different photoperiods, to determine the molecular mechanism of the photoperiod regulating follicle development in the seasonal reproduction of animals. We found that, under different photoperiods, the synergistic effect of hormones in the HPO axis ultimately affects the FSH secretion, participates in the regulation of the Nodal/ALK7 signaling pathway, and regulates the granulosa cell apoptosis, thus affecting the ovarian function, and ultimately, seasonal reproduction. These results suggest that partial molecular mechanisms of the seasonal reproduction and provide a theory basis to manipulate the animal reproduction.

**Abstract:**

The photoperiod regulates the seasonal reproduction of mammals by affecting the follicle development, for which the granulosa cells provide nutrition. However, the underlying mechanism remains unclear. Here, Djungarian hamsters (*Phodopus sungorus*) were raised under different photoperiods to study the ovarian status and explore the potential mechanism of the follicle development mediated by the FSH-Nodal/ALK7 signaling pathway. Compared with the moderate daylight (MD) group, the short daylight (SD) group exhibited a significant decrease in the ovarian weight and increase in the atretic follicle number and granulosa cell apoptosis, whereas the long daylight (LD) group showed an increase in the ovarian weight, the growing follicle number, and the antral follicle number, but a decrease in the granulosa cell apoptosis. Based on these findings, the key genes of the Nodal/ALK7 signaling pathway controlling the granulosa cell apoptosis were studied using the quantitative real-time polymerase chain reaction and western blotting. In the SD group, the follicle-stimulating hormone (FSH) concentration significantly decreased and the Nodal/ALK7/Smad signaling pathways were activated, while the phosphatidylinositol 3-kinase (PIK3)/Akt signaling pathway was inhibited. The BAX expression was significantly increased, while the Bcl-xL expression was significantly decreased, leading to an increase in the caspase-3 activity, the granulosa cell apoptosis, and ovarian degeneration. However, in the LD group, the FSH concentration significantly increased, the Nodal/ALK7/Smad signaling pathway was inhibited, and the PIK3/Akt signaling pathway was activated. Taken together, our results indicate that the photoperiod can regulate the apoptosis of the granulosa cells by regulating the concentration of FSH, activating or inhibiting the Nodal/ALK7 signaling pathway, thereby affecting the ovarian function. Our research provides an important theoretical basis for understanding the photoperiod-regulated mechanisms of the mammalian seasonal reproduction.

## 1. Introduction

To adapt to changing environments, many temperate species adjust their reproductive activity to occur during specific periods. The photoperiod, one of the most significant environmental factors, can influence the seasonal reproduction [1], especially via the regulation of the hypothalamus–pituitary–ovary (HPO) axis [2]. Following the elucidation of the endocrine mechanism of the HPO axis, the granulosa cells have become the focus of many studies [3] and are crucial for the follicle development and ovarian maintenance [4]. According to reports, various factors associated with the granulosa cell apoptosis are crucial for controlling atresia and follicle development [5]. For example, gonadotropin regulates the granulosa cell apoptosis [6], and insulin-like growth factor 1 (IGF-1) is necessary for the follicle development and the inhibition of the granulosa cell apoptosis. In addition, the secretion of the follicle-stimulating hormone (FSH) and luteinizing hormone (LH) is triggered by the gonadotropin-releasing hormone (GnRH) derived from the hypothalamus, which controls the apoptosis and the survival of the granulosa cells through the HPO axis, thus controlling the follicle development [7]. Previous research has shown that the photoperiod can regulate the granulosa cells in bank voles (*Myodes glareolus*) [8]. However, research on the effects of the photoperiod on the granulosa cell-related hormones and the mechanism underlying the granulosa cell apoptosis remains limited.

Nodal is an important growth factor, part of the transforming growth factor-β (TGF-β) superfamily [9], and is involved in regulating the granulosa cell apoptosis. Nodal serves biological functions through a combination with type I and type II receptor cell surface complexes [10], such as the activin-receptor-like kinase 7 (ALK7/ACVR1C), a type I receptor [11]. Once Nodal is activated by a decrease in the FSH concentration, the granulosa cell apoptosis can occur [12]. Nodal activates SMAD2 and SMAD3 after binding to ALK7, then forms a complex with SMAD4 to down-regulate the expression of the X-linked inhibitor of the apoptosis protein (XIAP) [13,14] and phosphate-Akt [15]. As the final core molecule, SMAD4 is essential for the TGF-β signal transduction [16,17]. SMAD4 can also inhibit the porcine ovarian granulosa cell apoptosis [18]. Furthermore, the phosphatidylinositol 3-kinase (PI3K)/Akt pathway can crucially regulate the granulosa cell apoptosis [15]. Akt can inhibit XIAP [19] and increase the mitochondrial release of death proteins, such as DIABLO, HtrA2, and cytochrome C [20,21], which promote the cell apoptosis. In the process of apoptosis, BAX can change the permeability of the outer mitochondrial membrane (OMM), which is part of the mitochondrial apoptosis pathway and can promote apoptosis. However, as an anti-apoptotic protein, Bcl-2 can inhibit apoptosis by inhibiting the release of the pro-apoptotic protein from mitochondria and inhibiting the pro-apoptotic protein BAX [22,23,24]. So, BAX and Bcl2 are important apoptotic molecules in mammalian mitochondria. The up-regulation of the BAX expression and down-regulation of the Bcl2 expression can promote the caspase-3 enzymatic activity [25,26,27]. In healthy follicles, the Nodal/ALK7 signaling pathway is inhibited under increased concentrations of the FSH, which combines with the FSH receptor (FSHR) to activate the PI3K/Akt signaling pathway, inhibit caspase-3, and increase the cell survival [12]. Therefore, the degree of the granulosa cell apoptosis may influence the ovarian function and the increased Nodal/ALK7 expression may contribute to the follicular standstill process [12]. Currently however, research on the effects of the photoperiod on the Nodal/ALK7 signaling pathway remains limited. A quantitative analysis of the key factors of the Nodal/ALK7 signaling pathway in the ovaries should help clarify the mechanism by which the photoperiod influences the granulosa cell apoptosis and ovarian function.

Djungarian hamsters (*Phodopus sungorus*), a typical seasonal reproductive animal [28], is widely distributed across the grasslands of Inner Mongolia, and are characterized as pests, due to their strong reproductive capacity. Thus, studying the effects of different photoperiods on the seasonal reproduction of *Phodopus sungorus* may provide new insights into rodent control. Here, the effects of different photoperiods on the ovarian structure and related mechanisms were studied in *Phodopus sungorus*. Notably, we explored: (1) the histological differences in the ovary under different photoperiods; (2) the differences in the concentrations of serum IGF-1, GnRH, FSH, and LH, under different photoperiods; (3) the differences in the granulosa cell apoptosis under different photoperiods; (4) the changes in the co-localization of Nodal and ACVR1C in the granulosa cells in the follicles under different photoperiods; (5) the differences in the mRNA and protein expression levels of the Nodal/ALK7 signaling pathway in the ovaries under different photoperiods; and (6) the changes in the caspase-3 activity under different photoperiods. The results of this study not only expand the current research regarding the molecular ecology of rodents, but also provide new insights into their reproductive mechanisms and a theoretical basis for the prevention and control of rodent pests in the grasslands.

## 2. Materials and Methods

### 2.1. Sample Collection and Treatments

*Phodopus sungorus* were captured in Xilinhot, Inner Mongolia, and prepared in our laboratory, following the procedure detailed in a previous study [29]. The captured hamsters were identified, numbered, and housed in a feeding room with natural light and composite rat food particles and water provided ad libitum. All experiments were reviewed and approved by the Biomedical Ethics Committee of Qufu Normal University (Permit Number: 2022052). All procedures followed the practice rules of the Chinese Experimental Animal Ethics Committee.

Sixty adult (2–3 months of age) female hamsters were selected by the estimation of the degree of molar wear, which is a common indicator to identify the age of mammals, mainly rodents [30,31,32,33]. The selected individuals were split into three groups at random, every group including 20 female hamsters. Additionally, according to the seasonal reproduction characteristics of *Phodopus sungorus* [34,35,36], three varied photoperiods were adopted, which were short daylight (light: darkness = 8 h: 16 h; SD), simulating a non-breeding season; long daylight (light: darkness = 16 h: 8 h; LD), mimicking a breeding season; and moderate daylight (light: darkness = 12 h: 12 h; MD) as a control. Then, the three groups were kept separately under SD, LD, and MD conditions at 22 ± 2 °C, 55% ± 5% RH, and a light intensity of 150 ± 10 lx for 6 weeks.

### 2.2. Sample Preparation

**Figure 1:**
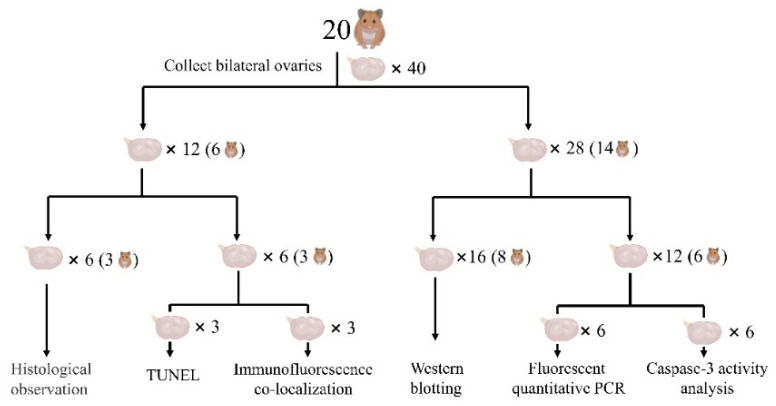
Ovarian experiment design. Note, the SD group was taken as an example.

The hamsters were asphyxiated by CO_2_ at 22:00 on the last day before the experiment after being kept in the dark for at least 2 h. Immediately afterwards, the fresh blood was collected and the serum was extracted. Serum IGF-1, GnRH, FSH, and LH concentrations were determined by the enzyme-linked immunosorbent assay (ELISA) (Shanghai Jining Biotechnology Co., Ltd., JN01331, JN07049, JN02022, JN00518, Shanghai, China). All ovaries were collected and weighed. The ovaries of six hamsters in each group were fixed with 4% paraformaldehyde phosphate-buffered fixation solution for the prepared paraffin and frozen-sections. The bilateral ovaries of three hamsters were used for the histological observation by hematoxylin and eosin (H&E) staining, while the left and right ovaries of the remaining three hamsters were used for terminal deoxynucleotidyl transferase biotin-dUTP nick end labeling (TUNEL) and immunofluorescence co-localization, respectively. The remaining 14 hamsters in each group had their ovaries frozen in liquid nitrogen and then maintained at −80 °C. Among them, the bilateral ovaries of eight hamsters were used for western blotting, while the left and right ovaries of the other six hamsters were used for the fluorescent quantitative polymerase chain reaction (PCR) and the caspase-3 activity analysis, respectively. The ovarian experimental design is shown in Figure 1.

### 2.3. Histological Study

The histological variations in the follicles from the ovarian tissue were observed by H&E staining. First, the ovaries were embedded in paraffin blocks for sectioning (5 μm thick), then rehydrated for hematoxylin staining (3 min) and rinsed with tap water (15 min). The sections were then differentially stained using 1% hydrochloric acid–ethanol solution (15 s), washed with tap water (5 min), counterstained with eosin (5 min), washed with tap water (15 min), and sealed with neutral gum. Finally, the stained sections were imaged using an upright optical microscope (NIKON Eclipse ci, NIKON digital sight DS-FI2, Nikon, Japan). The ovarian structures were analyzed, according to the type of follicles: growing follicles with more than three layers of cubic granulosa cells and no antrum present [37]; antral follicles with multiple layers of granulosa cells and antrum present [38]; and atretic follicles, the morphology of which is complex (at least five pyknotic granulosa nuclei and/or degenerated oocytes; a highly vascularized luteinized cell surrounded by the pseudotheca, 210–430 μm in diameter; a highly vascularized luteinized cell capsule, 150–270 μm in diameter) [38,39]. Three sections per ovary were examined.

### 2.4. TUNEL Staining

The ovarian apoptotic levels were evaluated using a TUNEL kit (G1501, Servicebio, Wuhan, China) and in situ TUNEL technique. Firstly, the frozen sections were prepared following the manufacturer’s specifications. Following the dehydration of the fixed ovarian tissue, the optimal cutting temperature compound (OCT) embedding agent was dropped around the tissue, and the specimen chuck was moved to the quick-freezing table of the frozen section machine for rapid freezing and embedding. When the OCT became white and hard, it could be sectioned. The slice thickness was 8–10 μm, used for the subsequent TUNEL detection. Prior to the TUNEL detection, the frozen sections needed to be preprocessed. Firstly, the samples were placed in a 37 °C oven for 22 min. Subsequently, 4% paraformaldehyde was added to the samples (30 min). The samples were washed three times with PBS (pH = 7.4) for 5 min. A protease K working solution was added dropwise to the tissue, which was then incubated in a 37 °C incubator for 22 min. The tissue sections were set aside in a 37 °C flat wet box for 2 h, followed by 4′,6-diamidino-2-phenylindole (DAPI) counterstaining at room temperature. The sections were observed under an ortho-fluorescent microscope (Nikon Eclipse C1, Nikon DS-U3 imaging system, Nikon, Japan). Then, a semi-quantitative study of the apoptotic activity in the granulosa cells was performed, based on three sections per ovary. The specific methods are as follows. Three regions of 200 × 200 μm^2^ were randomly selected from each section at the same magnification to examine the number of apoptotic granulosa cells (Granulosa cells TUNEL +) and the normal granulosa cells (Granulosa cells TUNEL -). Then, calculating the apoptosis activity by the formula of granulosa cells TUNEL +/(Granulosa cells TUNEL + and -) × 100.

### 2.5. Immunofluorescence Co-Localization

The co-localization of Nodal and ACVR1C in the ovary was determined using standard double immunofluorescence [40]. Firstly, the frozen sections were prepared following the manufacturer’s specifications. Prior to the detection, the frozen sections needed to be preprocessed. Then, they were subjected to antigen repair with an EDTA antigen repair buffer solution (pH 8.0). Secondly, the slice was incubated in a 3% hydrogen peroxide solution for 25 min to block the endogenous peroxidase (at room temperature). Subsequently, the sections were initially incubated overnight (4 °C) with rabbit anti-Nodal (bs-12243R, Bioss, Beijing, China), then with goat anti-rabbit IgG that had been tagged with horseradish peroxidase (HRP) (GB23303, Servicebio, Wuhan, China); CY3-TSA (CY3-Tyramide signal amplification; G1223, Servicebio, Wuhan, China) was added, and then incubated for 10 min at room temperature. The sections were washed with TBST for 5 min three times. Rabbit anti-ACVR1C (12610-1-AP, Proteintech, Wuhan, China) was incubated overnight (4 °C), and goat anti-rabbit IgG that had been labeled with Alexa Fluor^®^ 488 (GB25303, Servicebio, Wuhan, China) (50 min at room temperature) were added (see Table S1 for specific information). Lastly, the ovarian sections were counterstained at room temperature with DAPI for observation using an ortho-fluorescent microscope (Nikon Eclipse C1, Nikon DS-U3 imaging system, Nikon, Japan).

### 2.6. Quantitative Real-Time PCR

The total RNA of the ovaries was extracted by TRIzol reagent (D9108A, TaKaRa, Dalian, China), following the manufacturer’s specifications [41]. The quality and integrity of the extracted RNA were determined, based on the A260/A280 ratio and agarose gel electrophoresis (AGE), respectively. Using TaKaRa reagent, the qualified RNA was reverse-transcribed into cDNA and frozen at −80 °C, for later use. The experiment was completed using a SYBR^®^ Green Premix HS qPCR Kit II (Accurate Biotechnology Co., Ltd., Hunan, China). The amplification efficiency of the gene-specific primers was between 90% and 110%, and the degree of fitting was more than 0.99. In order to reduce the error, each gene was repeated in triplicate, and the difference of the threshold cycle (Ct) value among each repeat was less than 0.5. The mRNA expression level of the target genes including *Nodal*, *ALK7*, *SMAD4*, *XIAP*, *Akt*, *HtrA2*, *DIABLO*, *Bcl-xL*, *Bcl-2,* and *BAX* were normalized to that of the housekeeping gene (*β-actin*) using the 2−^△△CT^ method [42]. The differences for the expression level of the same gene between individuals from different photoperiod conditions, were analyzed using Fisher’s least significant difference (LSD) back testing and a one-way analysis of variance (ANOVA). All primers are shown detailed in Table S2 for the specific information.

### 2.7. Western Blotting

The total protein of the ovaries was extracted using RIPA lysate (G2002-100ML, Servicebio, Wuhan, China), a protease inhibitor (G2007-1ML, Servicebio, Wuhan, China), and PMSF (G2008-1ML, Servicebio, Wuhan, China). Additionally, the supernatants were mixed with 1 × SDS loading buffer including 100 mM Tris (pH 6.8), 5% 2-β-mercaptoethanol, 5% glycerol, 4% sodium dodecyl sulfate (SDS) (G2075-1ML, Servicebio, Wuhan, China), and bromophenol blue. Then, the SDS–polyacrylamide gel electrophoresis (10% Laemmli gel with an 30% acrylamide/bisacrylamide of 29:1; AR0138, Boster, Wuhan, China) was performed. Additionally, we used an electro-transferred Bio-Rad semi-dry transfer system on the specific polyvinylidene fluoride membranes (0.45 μm pore size, G6015-0.45, Servicebio, Wuhan, China), in accordance with previously described protocols [43]. The membrane was sealed with 5% skimmed milk powder (1 h at 37 °C), then initially incubated overnight (4 °C) with different primary antibodies, including rabbit anti-Nodal (DF7791, Affinity Biosciences, Melbourne, Australia), anti-ACVR1C (12610-1-AP, Proteintech, Wuhan, China), anti-SMAD4 (10231-1-AP, Proteintech, Wuhan, China), anti-XIAP (AF6368, Affinity Biosciences, Melbourne, Australia), anti P-Akt (AF0016, Affinity Biosciences, Melbourne, Australia), anti-DIABLO (10434-1-AP, Proteintech, Wuhan, China), anti-HtrA2 (15775-1-AP, Proteintech, Wuhan, China), anti-BAX (50599-1-AP, Proteintech, Wuhan, China), anti-Bcl2 (26593-1-AP, Proteintech, Wuhan, China), anti-Bcl-xL (10783-1-AP, Proteintech, Wuhan, China), anti-β-actin (20536-1-AP, Proteintech, Wuhan, China), anti-GAPDH (10494-1-AP, Proteintech, Wuhan, China), and anti-alpha tubulin antibodies (11224-1-AP, Proteintech, Wuhan, China), then with the secondary antibodies (IRDye 800 CW goat anti-rabbit) (31460, Thermo Fisher Scientific, Waltham, MA, USA) for 2 h at 37 °C (see Table S3 for specific information). Following the incubation, the membranes were visualized with an Odyssey scanner (Bio-Rad, Hercules, CA, USA) and the blots were quantified using Image-Pro Plus 6.0 software.

### 2.8. Detection of the Caspase-3 Enzymatic Activity

The ovary caspase-3 activity was determined using a hamster cysteine protease-3 ELISA kit (Shanghai Hengyuan Biological Co., HB023-Hr, Shanghai, China), in accordance with the manufacturer’s protocols [44]. The sample was prepared, according to the following steps, and the fresh ovarian tissue was washed with precooled PBS (pH = 7.4) to remove the residual blood. Subsequently, the tissue was cut into pieces. The tissue was fully ground on ice, the homogenate was centrifuged at 5000× *g* for 5 min, and the supernatant was taken for the follow-up detection. Each sample absorbance was assessed at 450 nm.

### 2.9. Statistical Analyses

Shapiro–Wilk and Levene tests were performed to test the normality of the data and the homogeneity of the variances. Using SPSS 20.0 software, the serum hormone concentrations (IGF-1, GnRH, FSH, and LH), the number and indices of each type of follicles, the apoptosis activity in the granulosa cells, the mRNA and protein expression of the ovary (Nodal, ALK7, SMAD4, XIAP, P-Akt, Akt, HtrA2, DIABLO, Bcl-xL, Bcl-2, and BAX) and caspase-3 activity were analyzed using Fisher’s least significant difference (LSD) back testing and a one-way analysis of variance (ANOVA), respectively, and the data visualization was performed with GraphPad Prism v8. All of analyzed data are displayed as means ± standard error of the mean (SEM). The thresholds for the statistical significance were * *p* < 0.05 and ** *p* < 0.01.

## 3. Results

### 3.1. Differences in the Body (BW) and Ovarian Weight (OW) in Hamsters under Different Photoperiods

There were no significant differences in the BW of hamsters before and after the photoperiod treatment. The BW of hamsters in the SD group decreased (by 0.22%), whereas that of the hamsters in the MD and LD groups increased (by 1.65% and 2.9%, respectively), compared with that before treatment, although the differences were not significant (*p* > 0.05). However, the OW was significantly lower in the SD group (*p* < 0.05) and higher in the LD group (*p* > 0.05), compared with the MD group, while the OW and the OW/BW ratios were significantly higher in the LD group than in the SD group (*p* < 0.05, Table 1).

### 3.2. Differences in the Serum Hormone Concentrations of the Hamsters under Different Photoperiods

**Figure 2:**
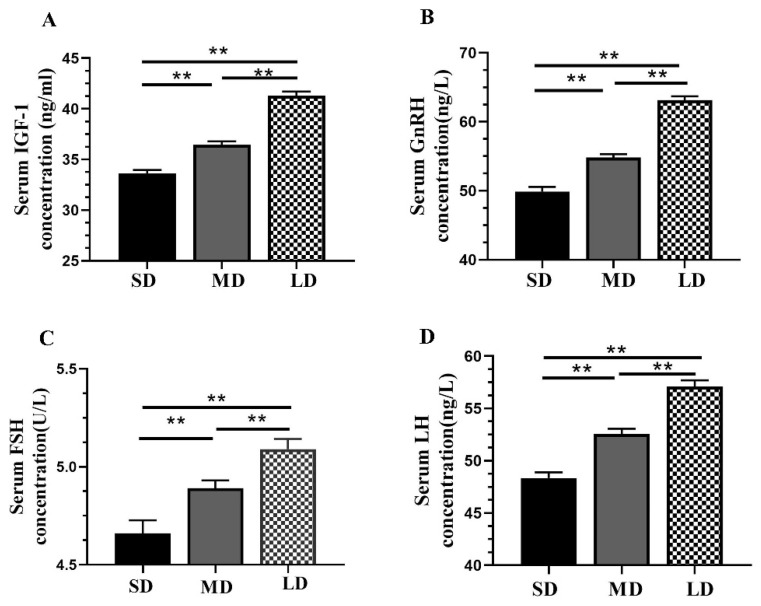
Differential analysis of the serum hormone concentrations in *Phodopus sungorus* under different photoperiod conditions. (**A**) IGF-1; (**B**) GnRH; (**C**) FSH; and (**D**) LH. Values are the means ± SEM. *n* = 11. SD, short daylight; MD, moderate daylight; LD, long daylight. ** *p* < 0.01.

The serum concentrations of the hormones, including IGF-1, GnRH, FSH, and LH, increased with the increasing illumination periods (Figure 2A–D), with significantly higher (*p* < 0.01) levels in the LD group and significantly lower levels in the SD group.

### 3.3. Differences in the Growing Follicles, Antral and Atretic Follicles in the Hamsters under Different Photoperiods

**Figure 3:**
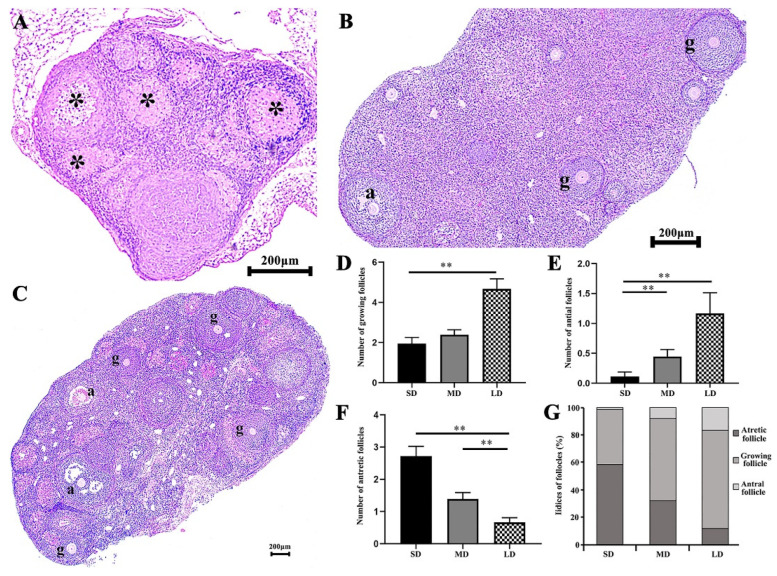
Differences in the number of follicles by the H&E staining of the ovaries of *Phodopus sungorus* under different photoperiods. (**A**) Ovarian section from the SD individuals. (**B**) Ovarian section from the MD individuals. (**C**) Ovarian section from the LD individuals. (**D**) Differences in the number of growing follicles across the different photoperiods. (**E**) Differences in the number of antral follicles across the different photoperiods. (**F**) Differences in the number of atretic follicles across the different photoperiods. (**G**) Indices of the growing follicles, antral and atretic follicles under the different photoperiods. g, growing follicle; a, antral follicle; *, atretic follicle. Values are means ± SEM. Bar = 200 μm; *n* = 3. SD, short daylight; MD, moderate daylight; LD, long daylight. * *p* < 0.05, ** *p* < 0.01.

The growing, antral, and atretic follicles are shown in Figure 3A–C, after photoperiod treatment. The ovaries showed an obvious degeneration in the SD group, with significantly more atretic follicles than in the LD group, and the MD group was significantly higher than the LD group (*p* < 0.01; Figure 3F, Table S6). The highest and lowest numbers of antral follicles were detected in the LD and SD groups, respectively; the number of antral follicles in the SD group was significantly lower than that in the MD and LD groups (*p* < 0.01; Figure 3E, Table S5). Additionally, there were significantly more growing follicles in the LD, compared with the SD (*p* < 0.01; Figure 3D, Table S4). At the same time, the indices of the atretic follicles, growing follicles, and antral follicles in the different photoperiods, were compared. It was found that the indices of the atretic follicles, growing follicles, and antral follicles in the SD group were 58.44%, 40%, and 1.56%, respectively. The proportions in the MD group were 31.83%, 60%, and 8.17%, respectively. The values for the LD group were 11.89%, 71.44%, and 16.67%, respectively (Figure 3G). Thus, the ovarian function was maintained in the LD conditions.

### 3.4. Apoptosis of the Granulosa Cells Examined by the TUNEL under Different Photoperiods

**Figure 4:**
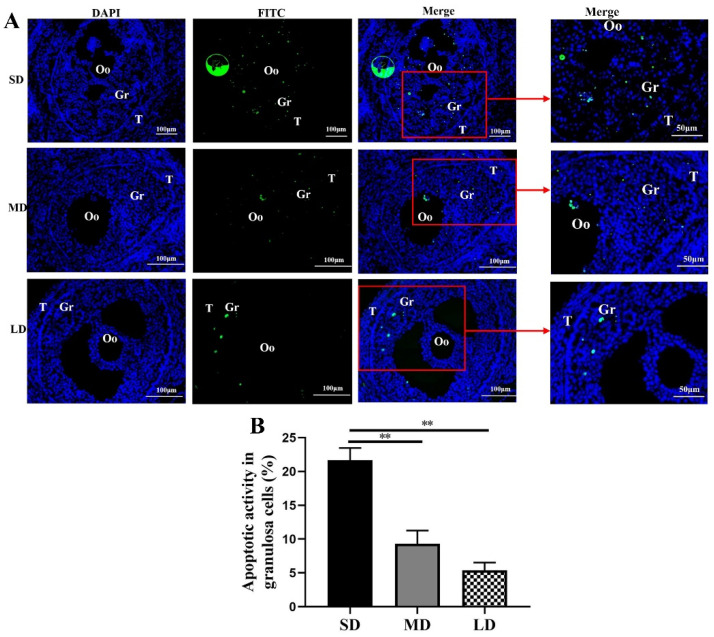
TUNEL detection results of the ovary tissue in *Phodopus sungorus* under three photoperiod conditions. (**A**) Fluorescent TUNEL staining of the ovaries of *Phodopus sungorus* under three photoperiod conditions. Blue, nuclei stained with 4′, 6-diamino-2-phenylindole (DAPI); green, TUNEL results of the FITC. The area indicated by the red arrow is an enlarged image of Merge. (**B**) Apoptosis activity of the granulosa cells in *Phodopus sungorus* under three photoperiod conditions. Bar = 100 μm; *n* = 3. Oo, oocyte; Gr, granulosa cells. SD, short daylight; MD, moderate daylight; LD, long daylight, ** *p* < 0.01.

The results of the TUNEL staining provided direct evidence of the cell apoptosis (Figure 4), with green fluorescence (indicating DNA fragmentation), mainly appearing in the granulosa cells. With the increase in the duration of light, the green fluorescence of the granulosa cells in the ovarian follicles decreased significantly. Significant differences in the granulosa cell apoptosis were observed across the different photoperiods. The highest and lowest levels of granulosa cell apoptosis were detected in the SD and LD groups, respectively, and the apoptosis activity of the granulosa cells in the SD group was significantly higher than that in the MD and LD groups (*p* < 0.01), indicating that the LD was the most beneficial photoperiod for the follicle development.

### 3.5. Interactions between Nodal and ACVR1C under Different Photoperiods

**Figure 5:**
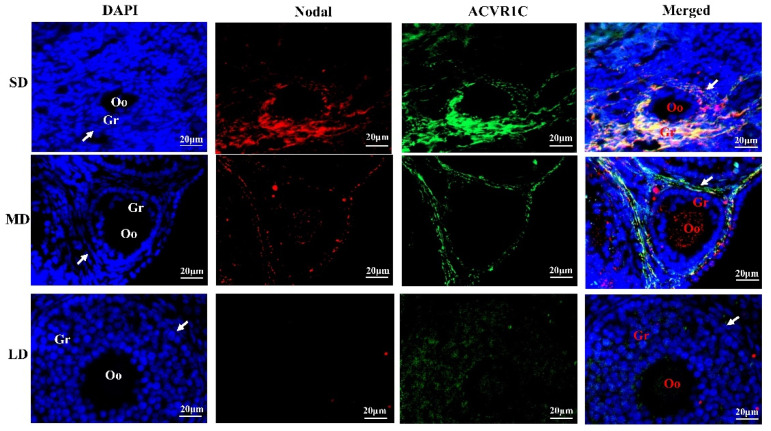
Co-localization of Nodal and ACVR1C proteins in the ovaries of *Phodopus sungorus* under three photoperiod conditions. Blue, nuclei stained with 4′, 6-diamino-2-phenylindole (DAPI); red, expression and distribution of Nodal protein; green, expression and distribution of ACVR1C protein. Oo, oocyte; Gr, granulosa cells; arrow shows theca cells. Bar = 20 μm; *n* = 3. SD, short daylight; MD, moderate daylight; LD, long daylight.

The immunofluorescence of the Nodal and ACVR1C co-localization in the follicles is shown in Figure 5. In the SD group, Nodal and ACVR1C were mainly co-localized in the granulosa cells, and the degree of co-localization was significantly increased, indicating that the interactions between Nodal and ACVR1C were enhanced. In the LD group, however, the interactions between Nodal and ACVR1C were inhibited. These results may explain the reason for the ovarian degeneration in the SD group but the ovarian function was maintained in the LD group.

### 3.6. Differentiated Expression of the Key Genes at the mRNA Level in the Nodal/ALK7 Signaling Pathway

**Figure 6:**
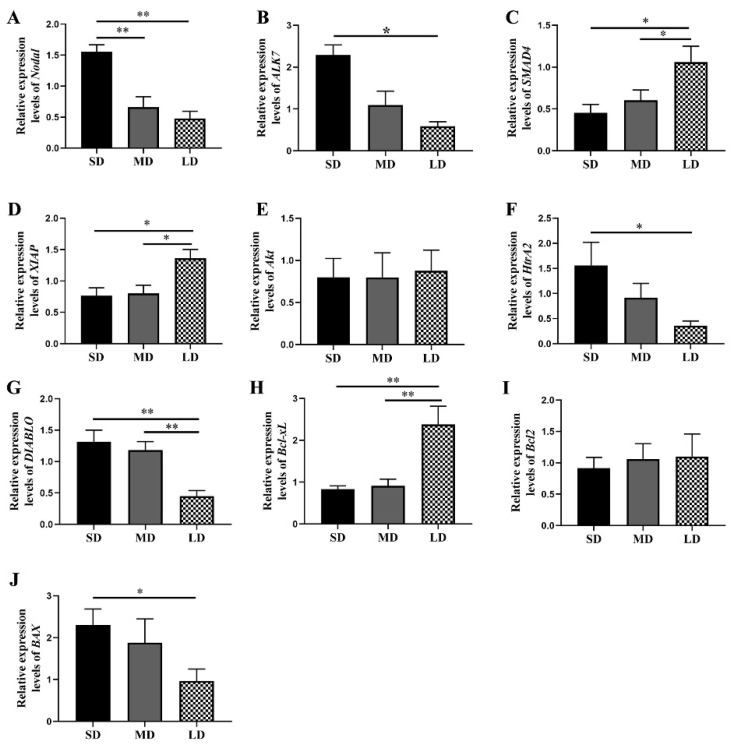
Key gene expression levels in the *Nodal/ALK7* signaling pathway in the ovaries of *Phodopus sungorus* under three photoperiod conditions. (**A**) *Nodal*, (**B**) *ALK7*, (**C**) *SMAD4*, (**D**) *XIAP*, (**E**) *Akt*, (**F**) *HtrA2*, (**G**) *DIABLO*, (**H**) *Bcl-xL*, (**I**) *Bcl2*, and (**J**) *BAX*. Values are means ± SEM. *n* = 6. SD, short daylight; MD, moderate daylight; LD, long daylight. * *p* < 0.05, ** *p* < 0.01.

Compared with the MD and LD groups, the *Nodal* expression was higher in the SD group (*p* < 0.01), whereas the *ALK7* expression was significantly higher in the SD group, compared with the LD group (*p* < 0.05; Figure 6A,B). Furthermore, compared with the MD and SD groups, the levels of the *SMAD4* and *XIAP* expressions were markedly increased in the LD group (*p* < 0.05; Figure 6C,D).

Our findings further suggested that the *Akt* expression followed the order LD > MD > SD (*p* > 0.05; Figure 6E). In addition, compared with the LD group, the *HtrA2* expression was markedly increased in the SD group (*p* < 0.05), while the *DIABLO* expression was significantly decreased in the LD group, compared with the other two groups (*p* < 0.01; Figure 6F,G).

The *Bcl-xL* expression levels were significantly enhanced in the LD group (*p* < 0.01; Figure 6H), while the *Bcl2* expression followed the order SD < MD < LD (*p* > 0.05; Figure 6I) and the *BAX* expression showed markedly higher levels in the SD group than in the LD group (*p* < 0.05; Figure 6J).

### 3.7. Differentiated Expression of the Key Genes at the Protein Level in the Nodal/ALK7 Signaling Pathway

**Figure 7:**
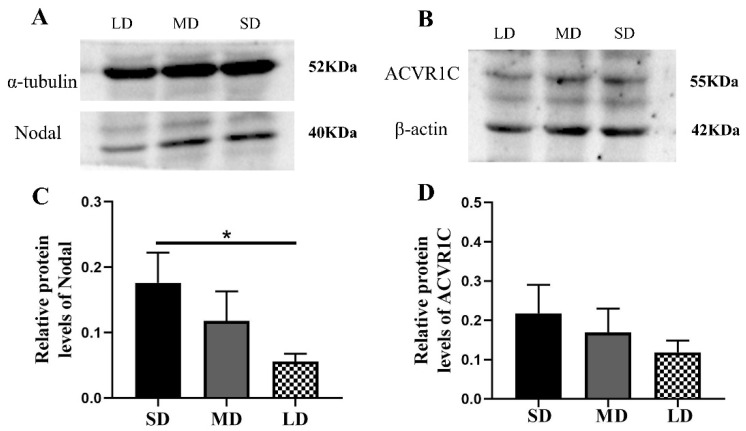
Levels of the Nodal and ACVR1C protein expression in the ovaries of *Phodopus sungorus* under three photoperiod conditions. (**A**,**B**) Representative immunoblots of the key Nodal and ACVR1C proteins and internal reference proteins (α-tubulin and β-actin) in three different photoperiod groups. (**C**,**D**) Relative protein expression of Nodal and ACVR1C. Values are means ± SEM. *n* = 8. SD, short daylight; MD, moderate daylight; LD, long daylight. * *p* < 0.05.

**Figure 8:**
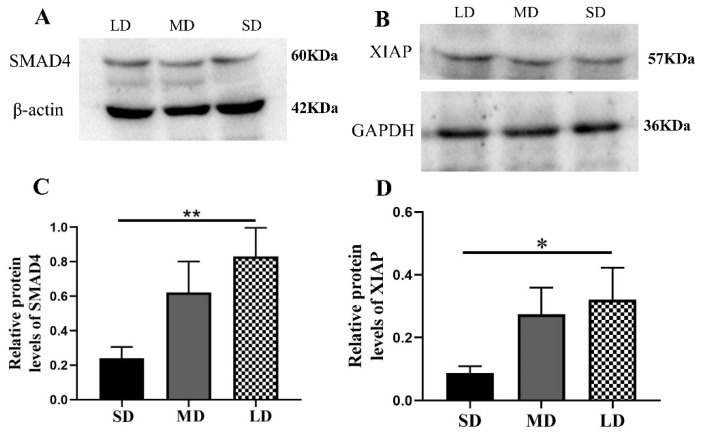
Levels of the Smad signaling pathway protein expression in the ovaries of *Phodopus sungorus* under three photoperiod conditions. (**A**,**B**) Representative immunoblots of the key SMAD4 and XIAP proteins and internal reference proteins (β-actin and GAPDH). (**C**,**D**) Relative protein expression of SMAD4 and XIAP. Values are means ± SEM. *n* = 8. SD, short daylight; MD, moderate daylight; LD, long daylight. * *p* < 0.05, ** *p* < 0.01.

Compared with the LD group, the Nodal expression was significantly increased in the SD group (*p* < 0.05) and the ACVR1C expression followed the order SD > MD > LD (*p* > 0.05; Figure 7A–D), whereas the SMAD4 (*p* < 0.01) and XIAP expressions (*p* < 0.05) were contrasting (Figure 8A–D).

**Figure 9:**
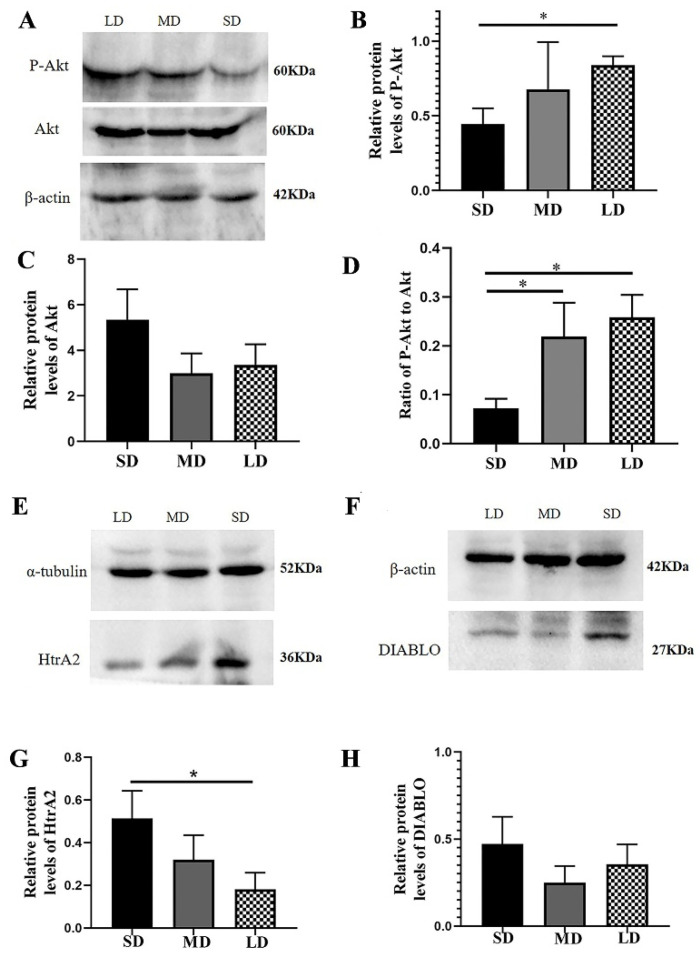
Levels of the PI3K/Akt signal pathway protein expression in the ovaries of *Phodopus sungorus* under three photoperiod conditions. (**A**,**E**,**F**) Representative immunoblots of the key P-Akt, Akt, HtrA2, and DIABLO proteins and internal reference proteins (β-actin and α-tubulin). (**B**,**C**,**G**,**H**) Relative protein expression of P-Akt, Akt, HtrA2, and DIABLO. (**D**) Ratio of P-Akt to Akt. Values are means ± SEM. *n* = 8. SD, short daylight; MD, moderate daylight; LD, long daylight. * *p* < 0.05.

The protein expression of P-Akt was clearly higher in the LD group than in the SD group (*p* < 0.05), although there were no significant differences in the Akt expression among the different groups (*p* > 0.05). The P-Akt to Akt ratio was substantially decreased in the SD group, compared with the LD and MD groups (*p* < 0.05; Figure 9A–D). In addition, compared with the LD group, the HtrA2 protein expression was significantly higher in the SD group (*p* < 0.05), although there was no significant difference in the DIABLO expression among the different groups (*p* > 0.05, Figure 9E–H).

**Figure 10:**
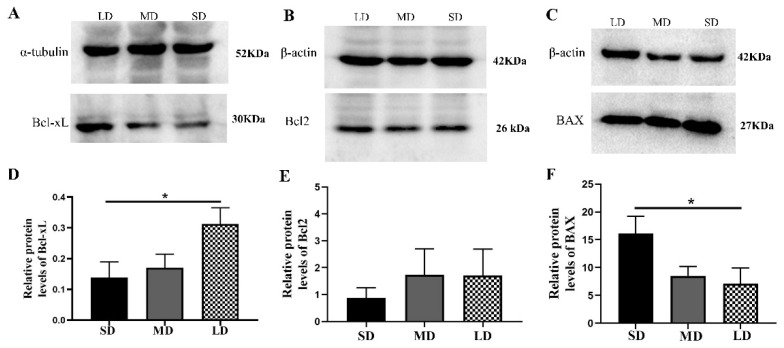
Levels of the B-cell lymphoma-2 (BCL-2) family protein expression in the ovaries of *Phodopus sungorus* under three photoperiod conditions. (**A**–**C**) Representative immunoblots of the key Bcl-xL, Bcl2, and BAX proteins and internal reference proteins (α-tubulin and β-actin). (**D**–**F**) Relative protein expression of Bcl-xL, Bcl2, and BAX. Values are means ± SEM. *n* = 8. SD, short daylight; MD, moderate daylight; LD, long daylight. * *p* < 0.05.

The results further showed that the Bcl-xL protein expression was significantly different between the SD and LD groups (*p* < 0.05; Figure 10A,D). The Bcl2 expression was also lower in the SD group, but not significantly different in the other groups (*p* > 0.05; Figure 10B,E). The BAX protein expression was notably increased in the SD group, compared with that in the LD group (*p* < 0.05; Figure 10C,F).

### 3.8. Differences in the Caspase -3 Enzyme Activity under the Different Photoperiods

**Figure 11:**
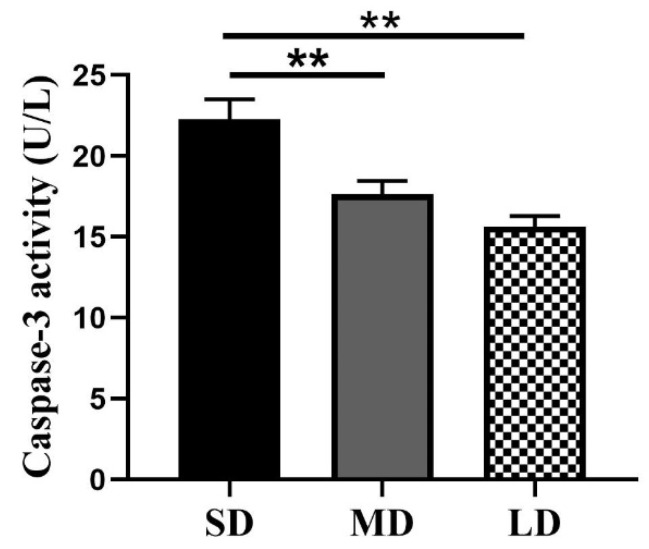
Detection of the caspase-3 activity in *Phodopus sungorus* under three different photoperiods. Values are means ± SEM. *n* = 6. SD, short daylight; MD, moderate daylight; LD, long daylight, ** *p* < 0.01.

The results of the caspase-3 activity are shown in Figure 11. The caspase-3 activity was highest in the SD group and lowest in the LD group, consistent with the TUNEL findings. In comparison with the MD and LD groups, the caspase-3 activity was considerably greater in the SD group (*p* < 0.01), but no significant differences were observed between the MD and LD groups (*p* > 0.05).

## 4. Discussion

**Figure 12:**
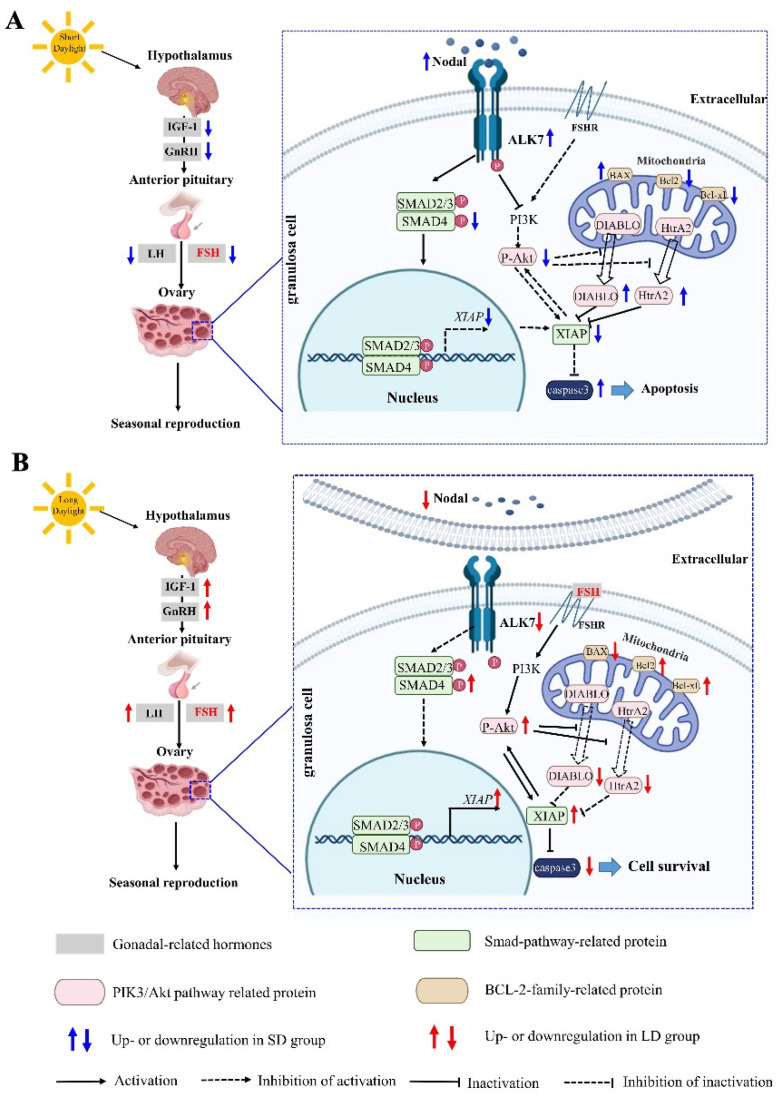
Molecular mechanism diagram of the photoperiod regulating the seasonal reproduction in female *Phodopus sungorus*. (**A**) Regulation mechanism of FSH-Nodal/ALK7 signal pathway under short daylight condition. (**B**) Regulation mechanism of FSH-Nodal/ALK7 signal pathway under long daylight condition. IGF-1: insulin growth factor 1; GnRH: gonadotropin-releasing hormone; FSH: follicle-stimulating hormone; LH: luteinizing hormone; ALK7: activin receptor-like kinase 7; BAX: bcl-2-associated X protein; Bcl-2: B-cell lymphoma-2; P-Akt: phosphate-Akt; HtrA2: High temperature requirement protein a2.

In this study, we explored the changes in the ovarian function and potential mechanisms of the molecular regulation under different photoperiods in *Phodopus sungorus*. Compared with the MD group, the SD group exhibited a significant decrease in the OW, as well as ovarian degeneration, while the LD group exhibited a significant increase in the OW, as well as ovarian function maintenance. Interestingly, the changes in the ovarian function in the different photoperiods were triggered by changes in the FSH concentration regulated by the HPO axis, resulting in the Nodal/ALK7 signaling pathway activation and the granulosa cell apoptosis in the SD group, with the opposite pattern observed in the LD group. These findings imply that changes in the ovarian function in different photoperiods are dependent on the degree of the granulosa cell apoptosis (Figure 12).

### 4.1. Hormone Synthesis and Ovarian Morphology Changes with the Photoperiods

The BWs of *Phodopus sungorus* were slightly greater in the LD and MD groups than in the SD and pre-treatment groups, after 6 weeks of various photoperiod treatments. This result is similar to previous reports on European hamsters (*Cricetus cricetus*), i.e., a short photoperiod can lead to weight loss [45]. In addition, the OW was significantly lower in the SD group than in the LD group, consistent with the photoperiod-induced weight loss associated with the loss of visceral organ mass, demonstrated in other species, such as Syrian hamsters (*Mesocricetus auratus*) [46]. These findings imply that the photoperiod may have a strong influence on the ovarian development and function in female *Phodopus sungorus*, with the decreasing OW in the SD group potentially weakening the reproductive capacity but the increasing OW in the LD group potentially enhancing the reproductive capacity. The reproductive characteristics of the female *Phodopus sungorus* are consistent with the phenomenon that long-day reproductive animals begin to reproduce in spring [47].

Importantly, we found significant differences in the ovarian tissue structure among the different groups. Based on the H&E staining, the SD group exhibited more atretic follicles and a higher degree of follicular atresia, as well as the pronounced deterioration of the ovaries. In contrast, the LD group showed a significant increase in growing and antral follicles, but a considerable decrease in atretic follicles. The follicular fate was determined by the granulosa cells’ developmental stage [48], which is essential for determining the follicle development [49,50,51,52]. Following the measuring of the different hormone concentrations in the different photoperiods, we found that, compared with the MD group, the serum concentration of the FSH was significantly lower in the SD group, whereas it was significantly higher in the LD group. This indicated that the changes in ovarian function were associated with changes in the hormone concentrations in the different photoperiods. As a cell survival factor [53] regulated by IGF-1, GnRH, and LH in the HPO axis, the FSH is crucial for inhibiting the granulosa cell apoptosis, promoting the follicle development [54,55,56], and regulating the ovarian development [57,58,59]. Here, compared with the MD group, the serum concentrations of IGF-1, GnRH, FSH, and LH were significantly lower in the SD group, but significantly higher in the LD group, indicating that the SD group promoted the granulosa cell apoptosis, whereas the LD group inhibited the granulosa cell apoptosis, as verified by the TUNEL assay. The changes in the FSH concentration caused by IGF-1, GnRH, and LH, in the HPO axis under different photoperiods may explain the degeneration of the ovaries in the SD group, but the maintenance of the ovarian function in the LD group. This indicates that the follicle development in the LD group was promoted by the increase in the FSH concentration; thus more follicles left the follicular pool for the development [60]. We speculate that the follicular pool in the ovary of *Phodopus sungorus* may have more opportunities to mature and successfully ovulate under LD conditions, resulting in increases in growing and antral follicles, and a decrease in atretic follicles, although this requires further verification.

The key factors (Nodal and ALK7) controlling the granulosa cell apoptosis were explored using the immunofluorescence co-localization. The results showed that the interactions between Nodal and ACVR1C were enhanced in the SD group, but weakened in the LD group, which may be the key reason for the ovarian degeneration in the SD group and the ovarian function maintenance in the LD group, which needs further confirmation. Both Nodal and ALK7 are hormonally regulated. With a decreasing FSH concentration, Nodal induces apoptosis by activating ALK7, but this interaction is inhibited under increasing FSH concentrations [12]. Therefore, we speculate that under SD conditions, interactions between Nodal and ALK7 are activated by a decrease in the FSH concentration, which further activates the downstream signaling pathway, thereby increasing the granulosa cell apoptosis and inhibiting the follicular growth, leading to an increase in follicular atresia and ovarian degeneration. Under LD conditions, the interactions between Nodal and ALK7 are inhibited by an increase in the FSH concentration, thereby inhibiting the granulosa cell apoptosis and promoting the follicular growth, leading to an increase in growing and antral follicles and the maintenance of the ovarian function.

### 4.2. Changes in the Nodal/ALK7 Signaling Pathway in the Ovary with the Different Photoperiods

The relationship between the Nodal/ALK7 signaling pathway and the FSH under different photoperiods remains unclear. Here, the gene expression levels related to the Nodal/ALK7 signaling pathway were measured under different photoperiods. The Nodal mRNA and protein expression levels increased significantly under the SD conditions, but decreased significantly in the LD group, consistent with earlier findings demonstrating that the relative *Nodal* abundance is significantly higher in the granulosa cells of atretic follicles than in healthy follicles, and the increase in the *Nodal* expression in the SD group may be a physiological signal that induces atresia [61]. The decreasing FSH is an important factor activating the interaction between Nodal and ALK7 [12]. Here, under the SD conditions, the concentration of FSH decreased and the interactions between Nodal and ALK7 were activated, further activating the Smad signaling pathway. The *SMAD4* expression is strongly correlated with the development of mammalian ovaries [62]. SMAD4 can also form a complex with SMAD2 and SMAD3 to inhibit the XIAP expression and induce follicular atresia [63]. This is in line with our findings, which showed that the expression levels of SMAD4 and XIAP were decreased and the follicular atresia was increased in the SD conditions. Under the LD conditions, however, the concentration of the FSH increased and the Nodal and ALK7 interactions were inhibited, thus inhibiting the Smad signal pathway. Furthermore, the FSH activated the PIK3/Akt signaling pathway by combining with the FSHR, thereby significantly increasing the expression of P-Akt. Akt and its downstream targets constitute another cell survival pathway [64], which is inhibited by Nodal to promote apoptosis. Consistently, our findings have demonstrated that the P-Akt expression was inhibited in the SD group. Akt promotes the cell survival and suppresses the apoptotic death by phosphorylating and stabilizing XIAP [19]. The mitochondrial DIABLO and HtrA2 can be released more readily when the Akt activity is inhibited [65,66]. This is also an important reason why the HtrA2 expression increased significantly in the SD conditions but decreased significantly in the LD conditions. Two pro-apoptotic proteins, i.e., DIABLO and HtrA2, released by the mitochondria, can specifically recognize and bind to XIAP [20,67,68,69,70], explaining why the XIAP expression increased in the LD group but decreased in the SD group. Proteins of the BCL-2 family are also essential for regulating the apoptosis of the granulosa cells [71]. As a pro-apoptotic factor, BAX can induce apoptosis by permeating the outer mitochondrial membrane and starting the caspase cascade, whereas Bcl-2 and Bcl-xL can inhibit apoptosis [72]. Under the SD conditions, the Bcl-xL expression levels were significantly decreased, whereas the BAX expression levels were significantly increased. Under the LD conditions, the Bcl-xL expression levels were significantly increased, whereas the BAX expression levels were significantly decreased. This is consistent with previous research demonstrating how SD can increase the expression of the apoptotic markers, including BAX, in the testes, causing organ atrophy [73,74,75]. Additionally, we discovered that the caspase-3 activity increased significantly in the SD conditions, with associated increases in the apoptosis, atresia follicles, and ovarian degeneration. In the LD conditions, the caspase-3 activity decreased significantly, with an associated decline in the granulosa cell apoptosis, increase in the cell survival, more growing follicles, and antral follicles, and a decrease in atretic follicles, thereby promoting the development of follicles and ovarian function. This was due to the change in the XIAP expression caused by the Smad and PI3K/Akt signaling pathways in different photoperiods. As the only post-mitochondrial apoptosis inhibitor protein in the mitochondria, XIAP can inhibit pro-apoptosis effects by combining with the caspase proteins [76,77,78,79]. Under the SD conditions, the combination of XIAP and caspase decreased, thus promoting the granulosa cell apoptosis, whereas under the LD conditions, the combination of XIAP and caspase increased, thus inhibiting the granulosa cell apoptosis. These findings suggest that the Nodal/ALK7 signaling pathway acts differently under different photoperiods. This study expands our understanding of the molecular mechanisms by which the photoperiod acts on the ovary, and provides a theoretical basis for the reproductive regulation in rodents.

## 5. Conclusions

In conclusion, we explored the functional changes and associated mechanisms of the ovary under different photoperiods. The results showed that the changes were caused by the regulation of the Nodal/ALK7 signaling pathway via the FSH concentrations. Changes in the ovarian function were intimately related to the photoperiod in female *Phodopus sungorus*. The photoperiod affects the hormone concentrations in the HPO axis, especially the FSH. Hormonal changes controlled the Nodal/ALK7 signaling pathway and affected the ovarian function. The FSH concentrations increased in the LD conditions, which inhibited the Nodal/ALK7/Smad signaling pathway. The FSH then combined with the FSHR to activate the PI3K/Akt signaling pathway, resulting in a decrease in the granulosa cell apoptosis, the promotion of the follicular development, the maintenance of the ovarian function, and the promotion of the seasonal reproduction, with the opposite trends in the SD conditions. Our findings not only expand our understanding of the molecular ecology of rodents, but also provide a basis to prevent and mitigate the occurrence of grassland rodent pests.

## Figures and Tables

**Table 1:** Differences in the body and ovarian weights among hamsters under different photoperiods.

Group		SD	MD	LD
Prior to the photoperiod	BW (g)	32.5750 ± 0.28982	32.4800 ± 0.35715	32.2200 ± 0.26937
Following the photoperiod	BW (g)	32.5050 ± 0.411640	33.0250 ± 0.25944	33.200 ± 0.22478
	OW (g)	0.0264 ± 0.00071	0.0283 ± 0.00059 *	0.02882 ± 0.00065 *
	OW/BW (mg/g)	0.8002 ± 0.02402	0.8579 ± 0.02045	0.8697 ± 0.02217 *

BW, body weight; OW, ovarian weight; SD, short daylight; MD, moderate daylight; LD, long daylight. Values are means ± SEM. *n* = 20. ∗ *p* < 0.05 compared with the SD.

## Data Availability

Data are available from the corresponding author upon reasonable request.

## Supplementary Materials

The following supporting information can be downloaded from: https://www.mdpi.com/article/10.3390/ani12243570/s1, Table S1: Detailed information of the immunofluorescence co-localization antibody. Table S2: Fluorescence quantitative primer sequences and related information. Table S3: Detailed information of the western blotting antibody. Table S4: Numbers of growing follicles in each section under different photoperiods, with the same magnification. Table S5: Numbers of antral follicles in each section under different photoperiods, with the same magnification. Table S6: Numbers of atretic follicles in each section under different photoperiods, with the same magnification.
