# Supplementary material for: The Photoperiod Regulates Granulosa Cell Apoptosis through the FSH-Nodal/ALK7 Signaling Pathway in Phodopus sungorus
Source: Animals (Basel). 2022 Dec 16;12(24):3570. doi: 10.3390/ani12243570 (PMC9774567; doi:10.3390/ani12243570)
Supplement: Supplementary file 1 [file animals-12-03570-s001.zip › animals-2017514-supplementary-minor/animals-2017514-original WB figures.pdf]

Original images of Western blotting.

This is the original images of Western blotting with 11 proteins among 8 different individual, n=8. Some polyvinylidene fluoride (PVDF) membranes were sliced during the experiment in accordance with the required molecular weight range to make it easier to incubated with different primary antibodies at once, and we made sure that the internal reference and the target protein were both on the same membrane.

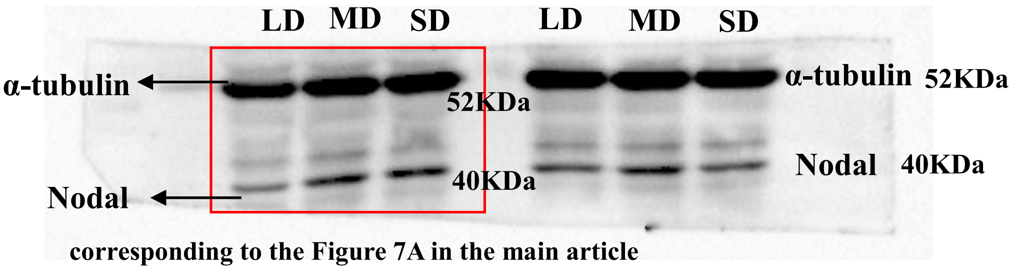

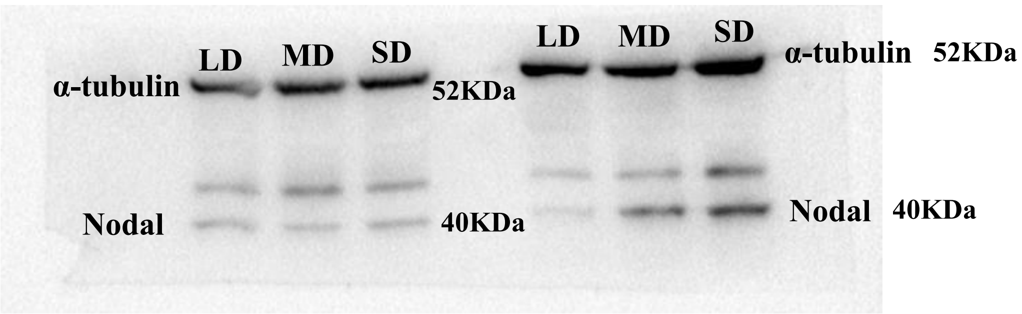

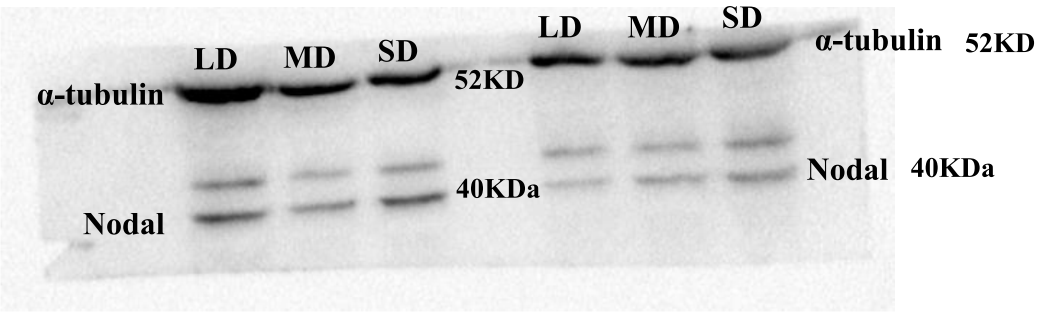

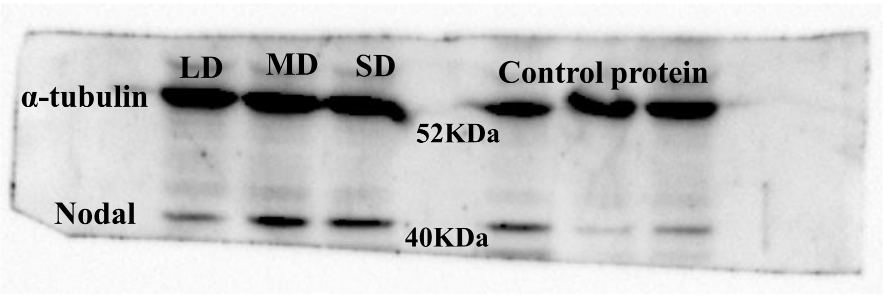

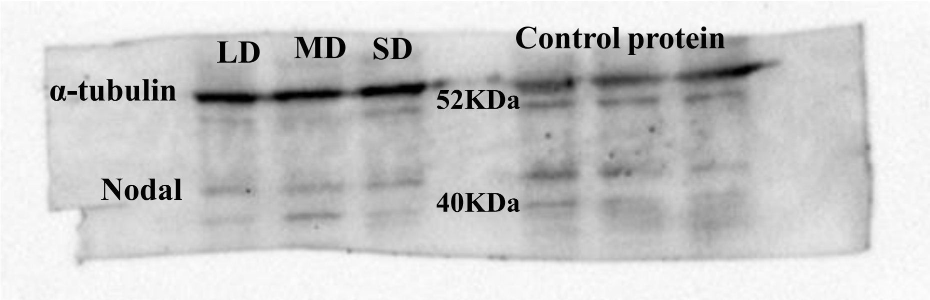
LD MD SD
Control protein
α-tubulin
52KDa
Nodal
40KDa

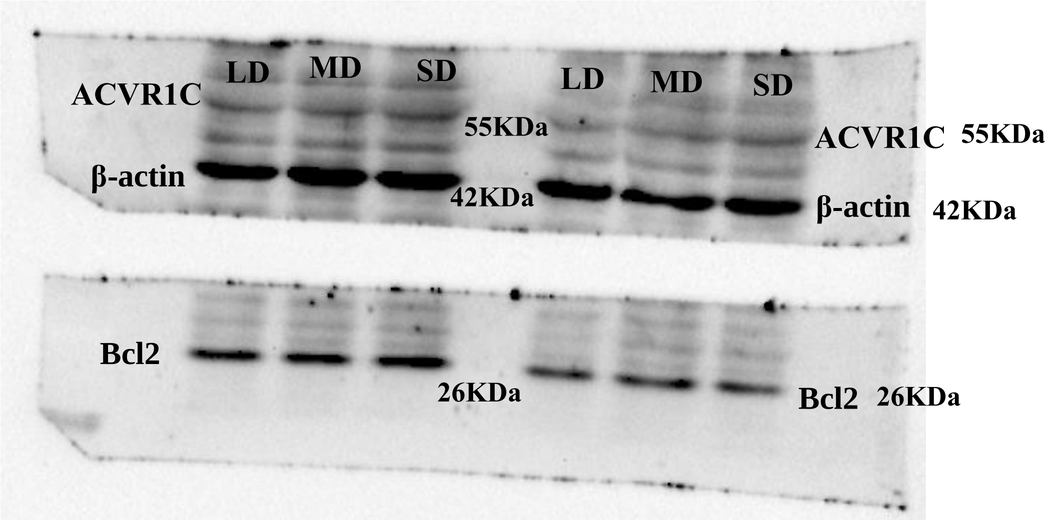
ACVR1C
LD MD SD
LD MD SD
55KDa
ACVR1C 55KDa
β-actin
42KDa
β-actin 42KDa
Bcl2
26KDa
Bcl2 26KDa

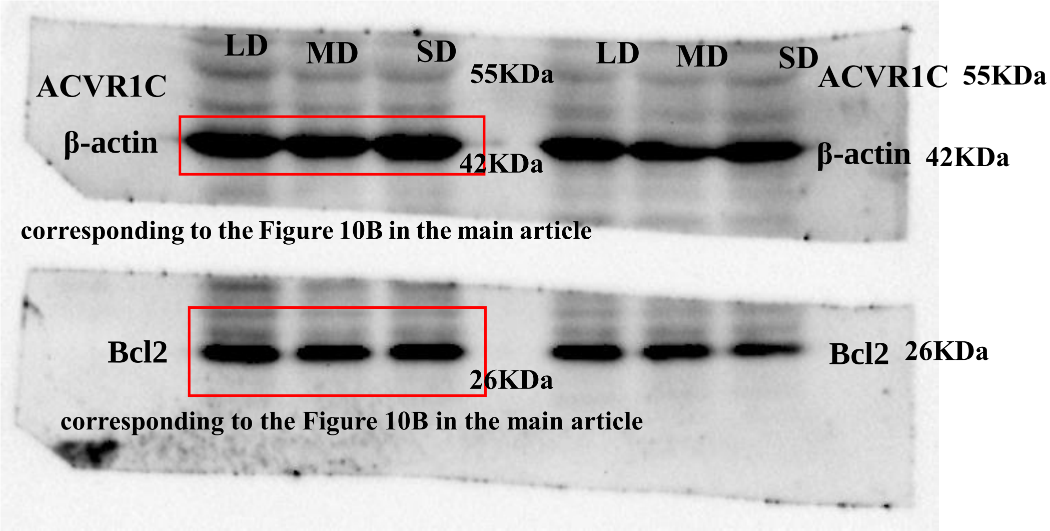
LD MD SD
LD MD SD
ACVR1C
55KDa
ACVR1C 55KDa
β-actin
42KDa
β-actin 42KDa
corresponding to the Figure 10B in the main article
Bcl2
26KDa
Bcl2 26KDa
corresponding to the Figure 10B in the main article

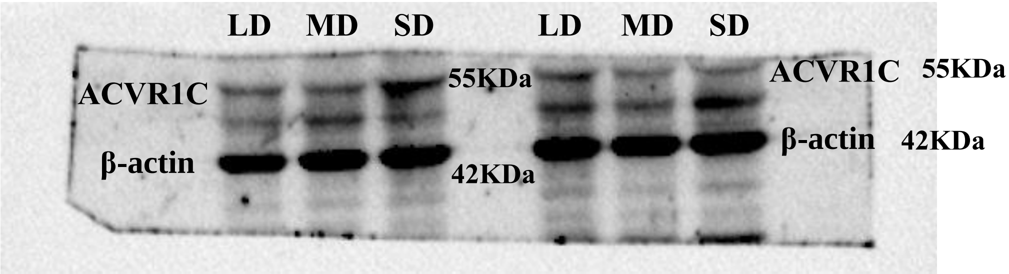
LD MD SD
LD MD SD
ACVR1C
55KDa
ACVR1C 55KDa
β-actin
42KDa
β-actin 42KDa

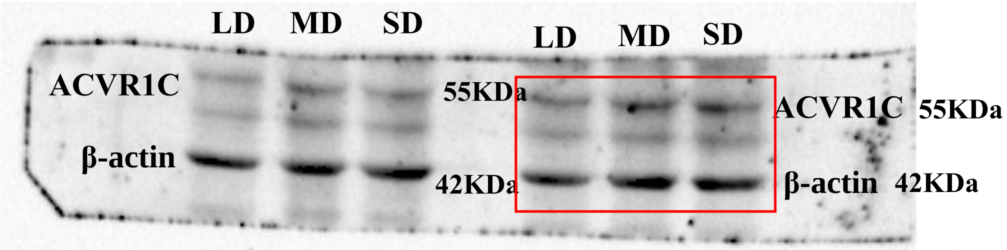

corresponding to the Figure 7B in the main article

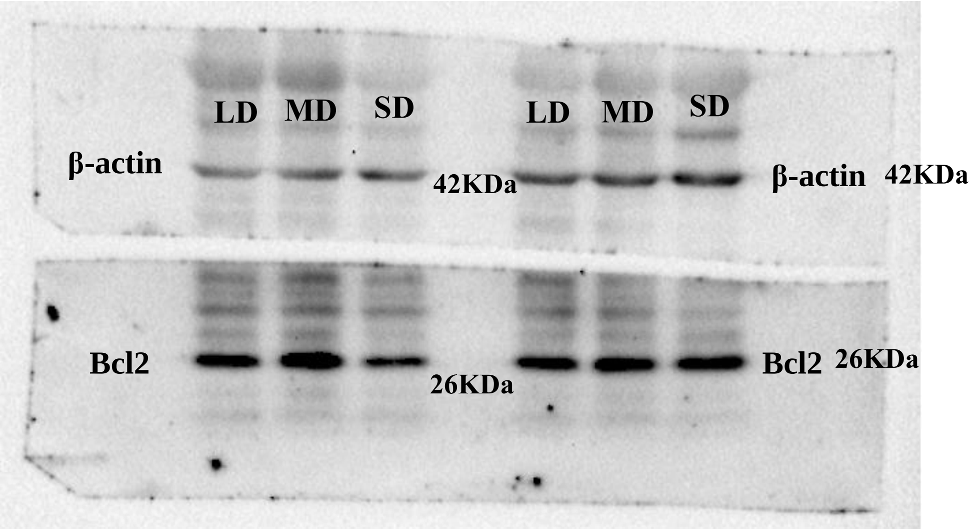

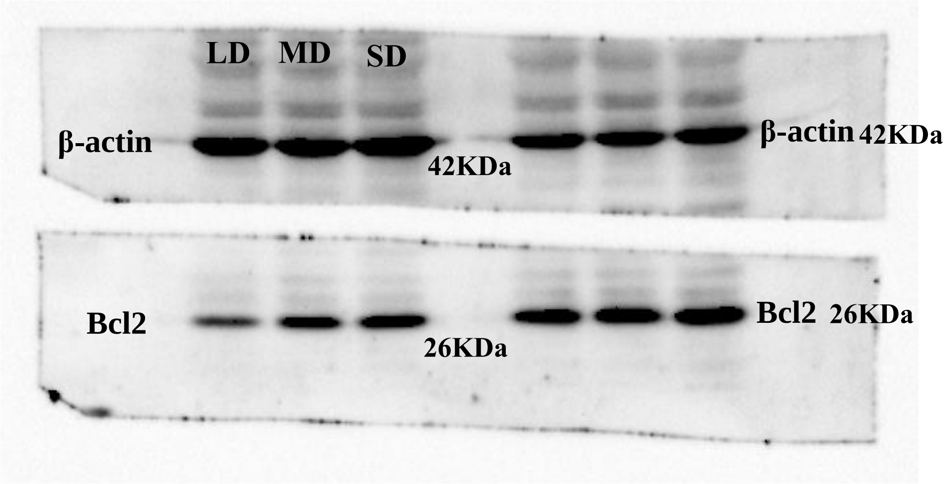

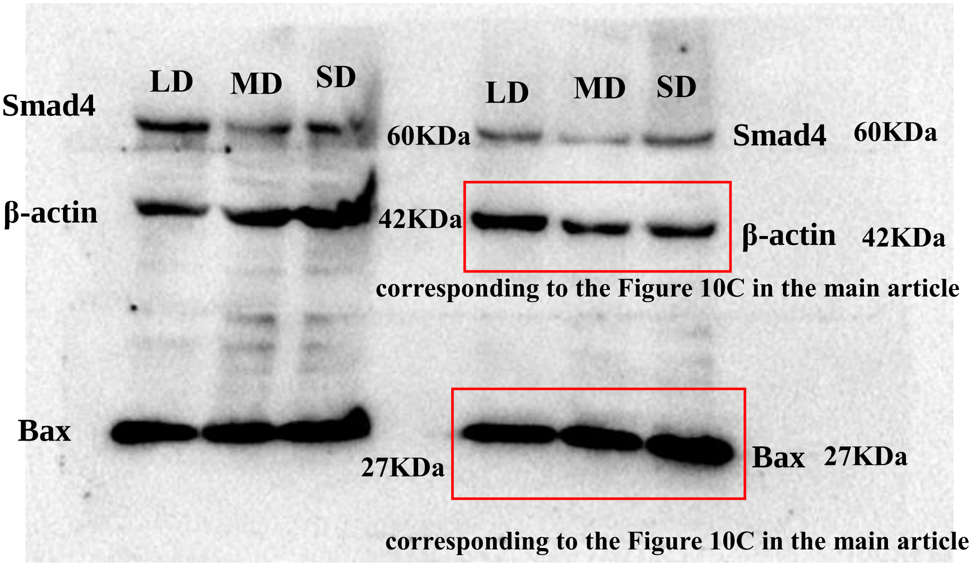
LD MD SD
LD MD SD
Smad4
60KDa
Smad4 60KDa
β-actin
42KDa
β-actin 42KDa
corresponding to the Figure 10C in the main article
Bax
27KDa
Bax 27KDa
corresponding to the Figure 10C in the main article

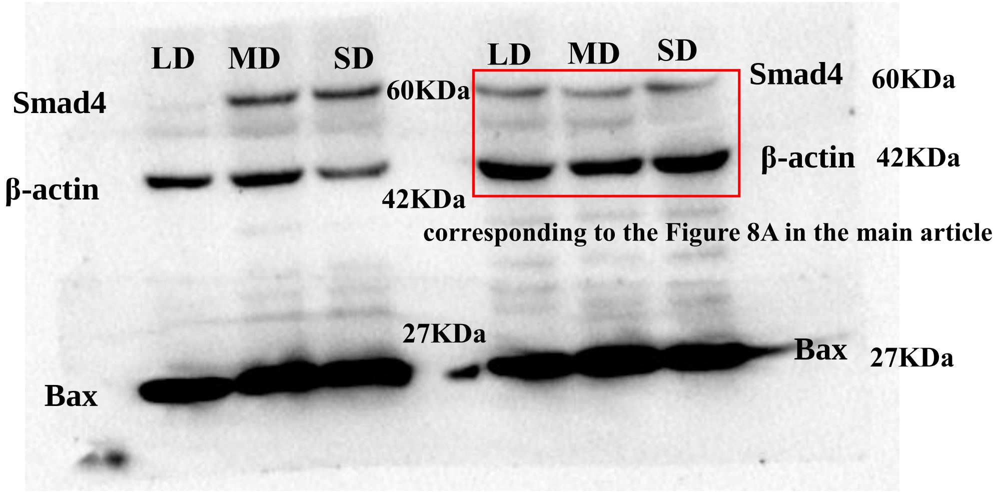
LD MD SD
LD MD SD
Smad4
60KDa
Smad4 60KDa
β-actin
42KDa
β-actin 42KDa
corresponding to the Figure 8A in the main article
27KDa
Bax 27KDa
Bax

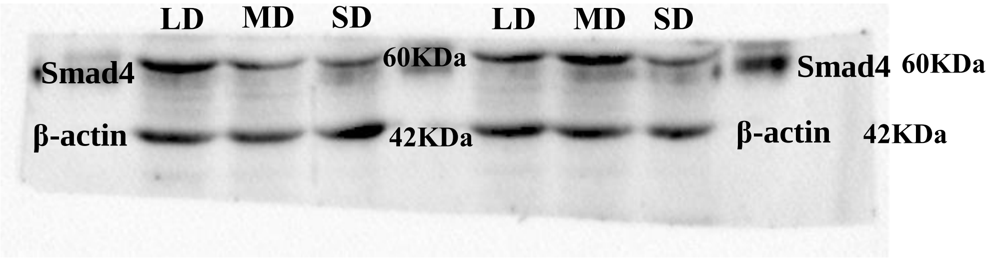
LD MD SD
LD MD SD
Smad4
60KDa
Smad4 60KDa
β-actin
42KDa
β-actin 42KDa

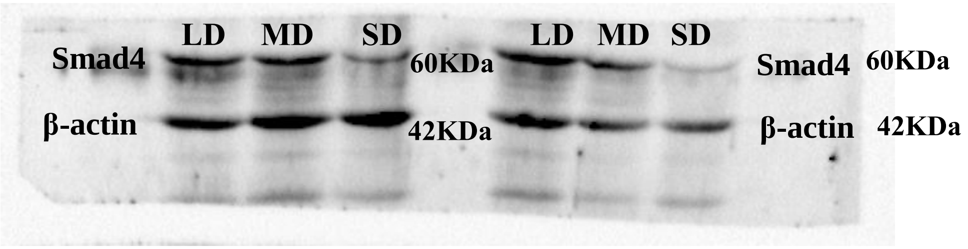
LD MD SD
LD MD SD
Smad4
60KDa
Smad4 60KDa
β-actin
42KDa
β-actin 42KDa

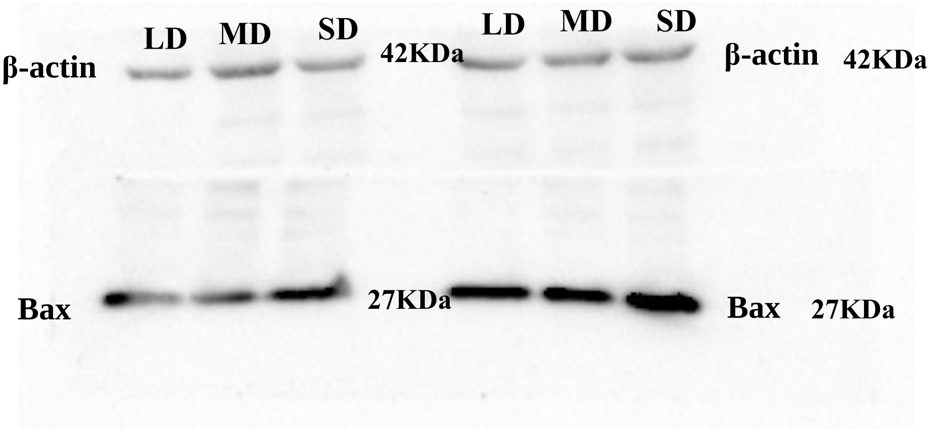

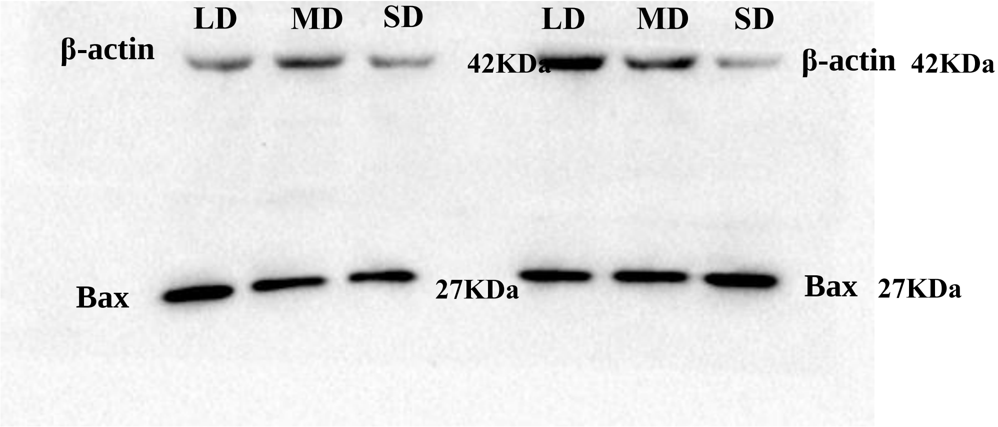

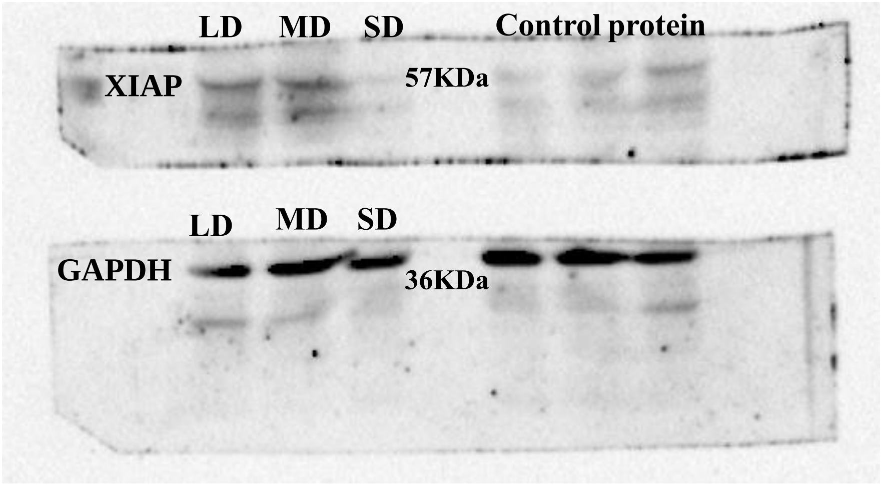

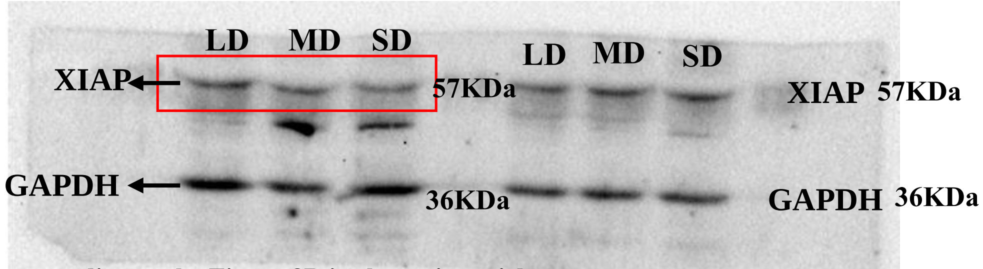

corresponding to the Figure 8B in the main article

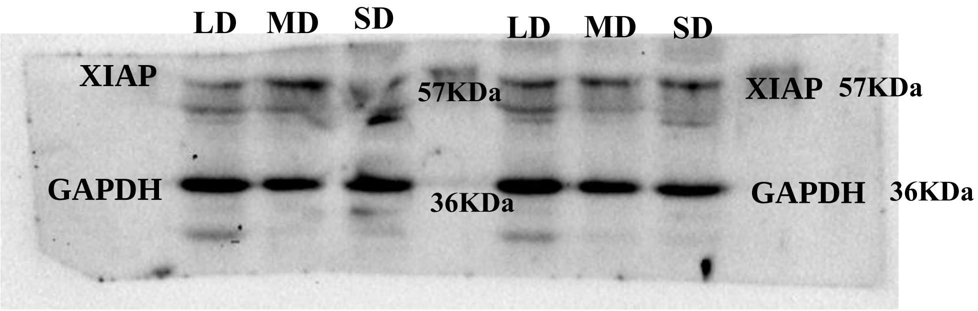

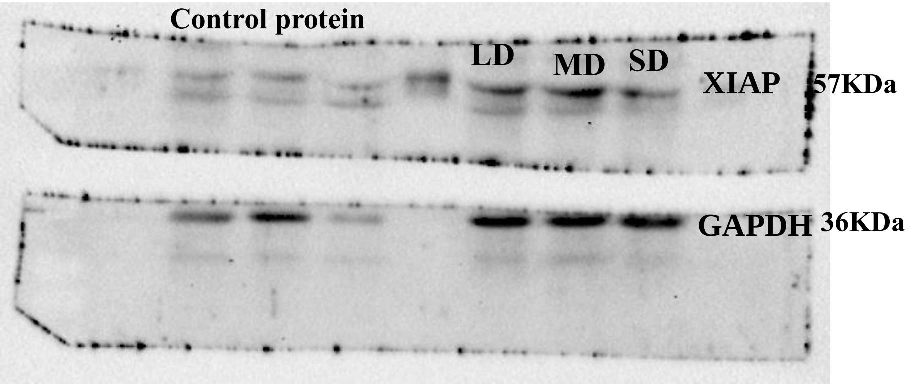

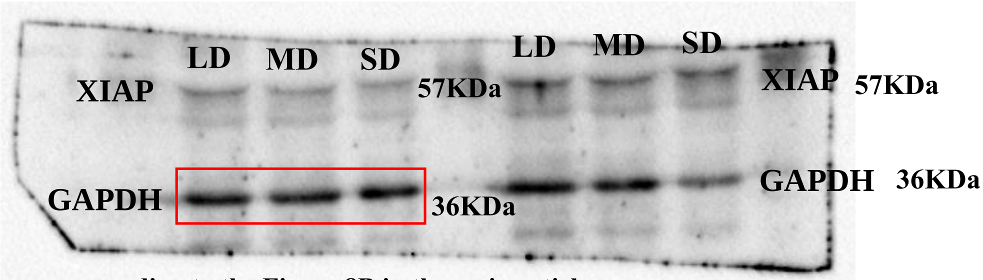

corresponding to the Figure 8B in the main article

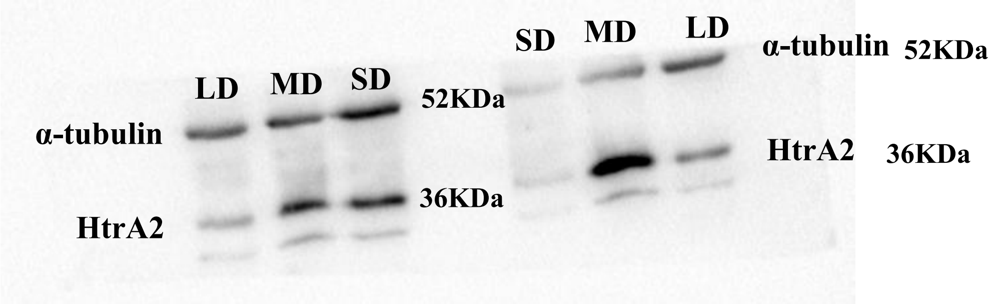

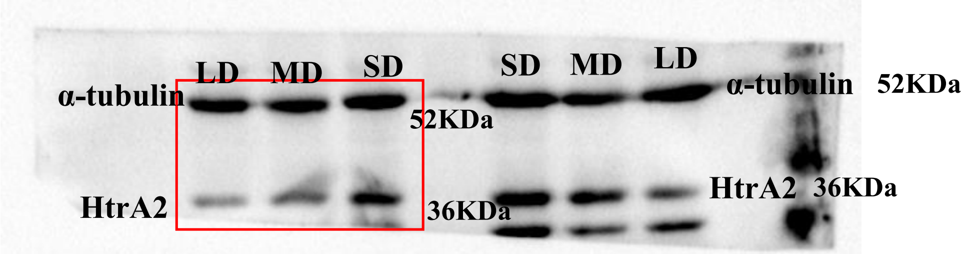

corresponding to the Figure 9E in the main article

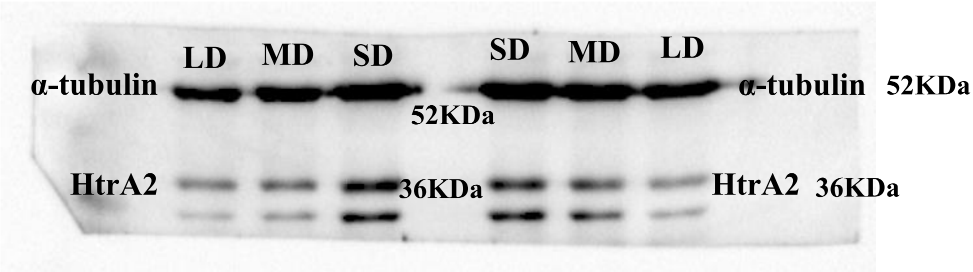
LD MD SD SD MD LD
α-tubulin
52KDa
α-tubulin 52KDa
HtrA2
36KDa
HtrA2 36KDa

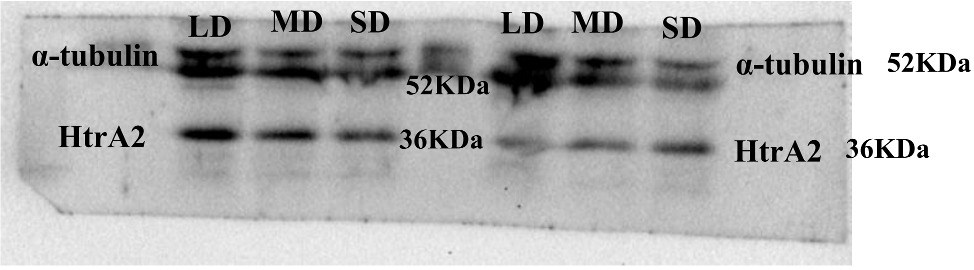
LD MD SD LD MD SD
α-tubulin
52KDa
α-tubulin 52KDa
HtrA2
36KDa
HtrA2 36KDa

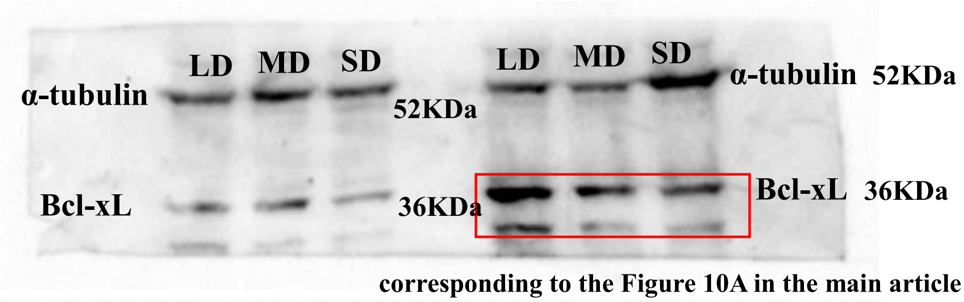
LD MD SD LD MD SD
α-tubulin
52KDa
α-tubulin 52KDa
Bcl-xL
36KDa
Bcl-xL 36KDa
corresponding to the Figure 10A in the main article

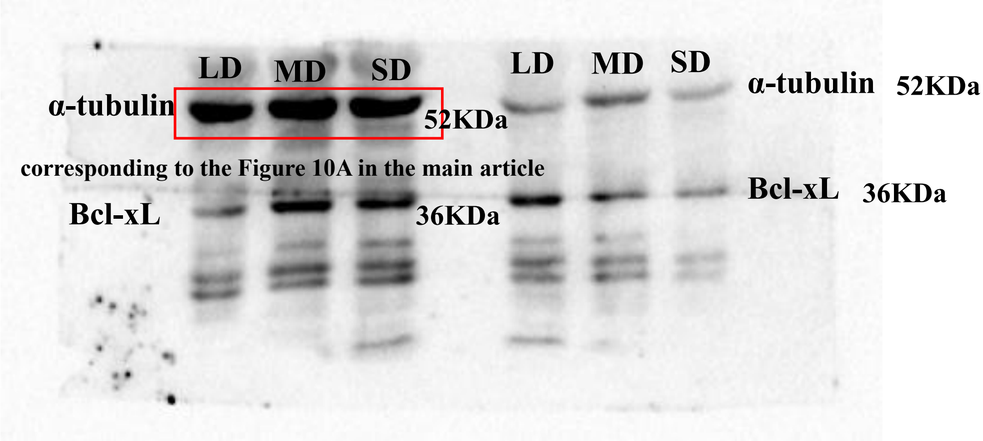
LD MD SD LD MD SD
α-tubulin
52KDa
α-tubulin 52KDa
corresponding to the Figure 10A in the main article
Bcl-xL
36KDa
Bcl-xL 36KDa

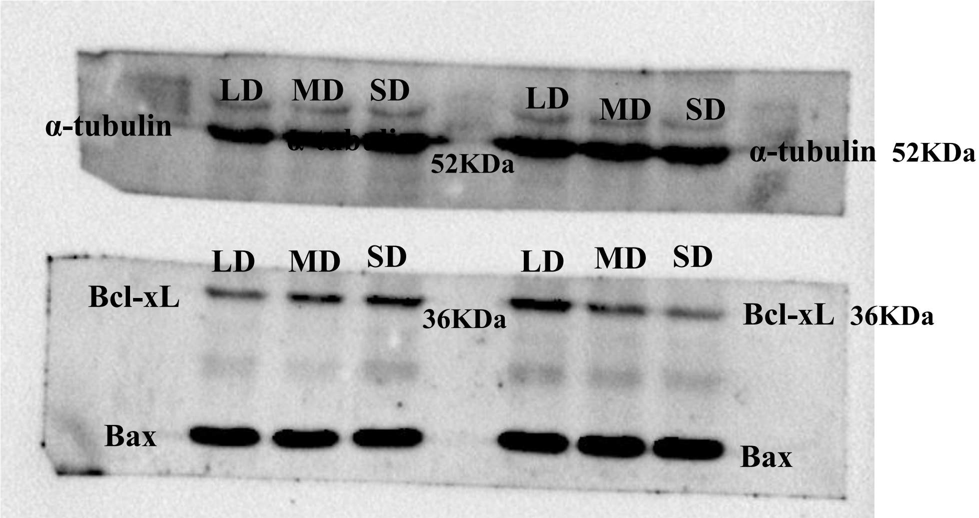
LD MD SD LD MD SD
α-tubulin
52KDa
α-tubulin 52KDa
LD MD SD LD MD SD
Bcl-xL
36KDa
Bcl-xL 36KDa
Bax
Bax

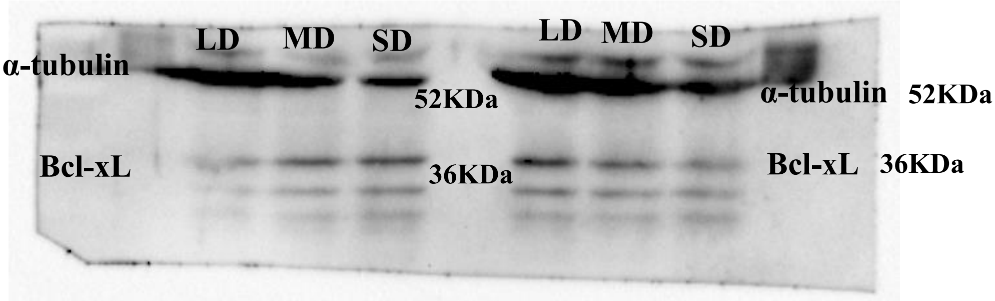
LD MD SD LD MD SD
α-tubulin
52KDa
α-tubulin 52KDa
Bcl-xL
36KDa
Bcl-xL 36KDa

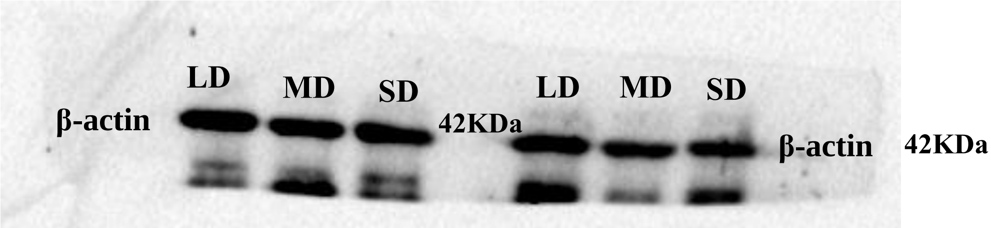
LD MD SD LD MD SD
β-actin
42KDa
β-actin 42KDa

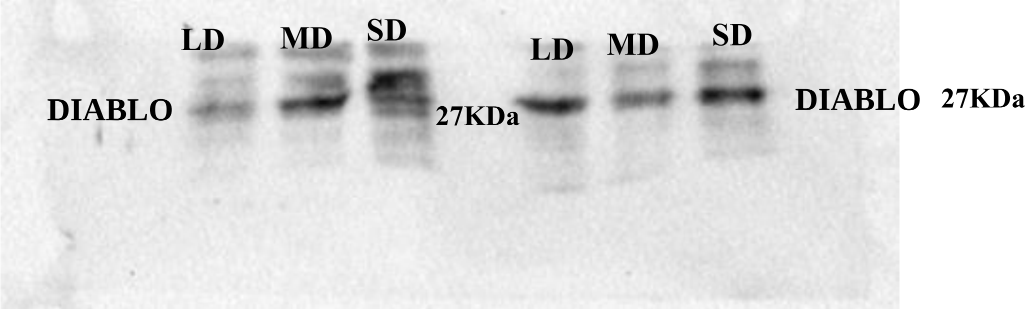
LD MD SD LD MD SD
DIABLO
27KDa
DIABLO 27KDa

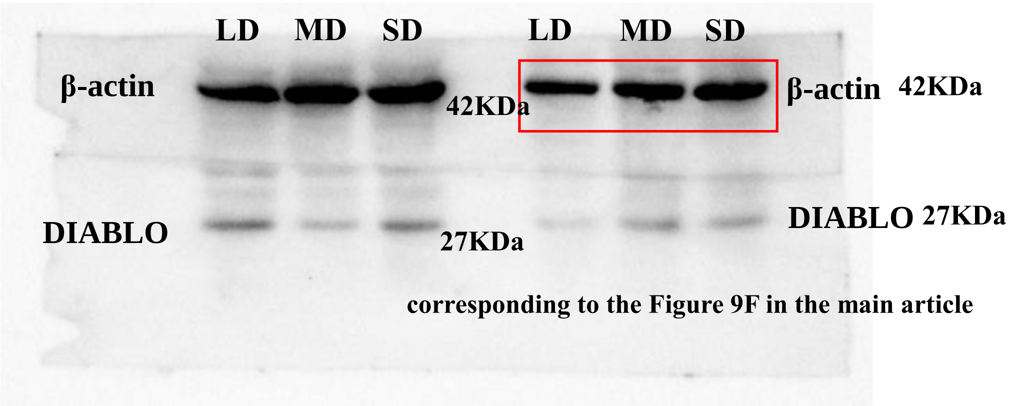

corresponding to the Figure 9F in the main article

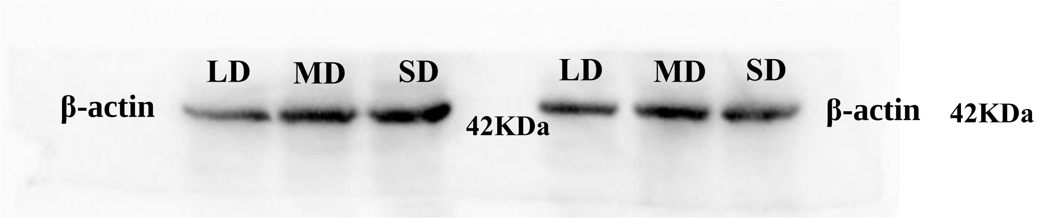

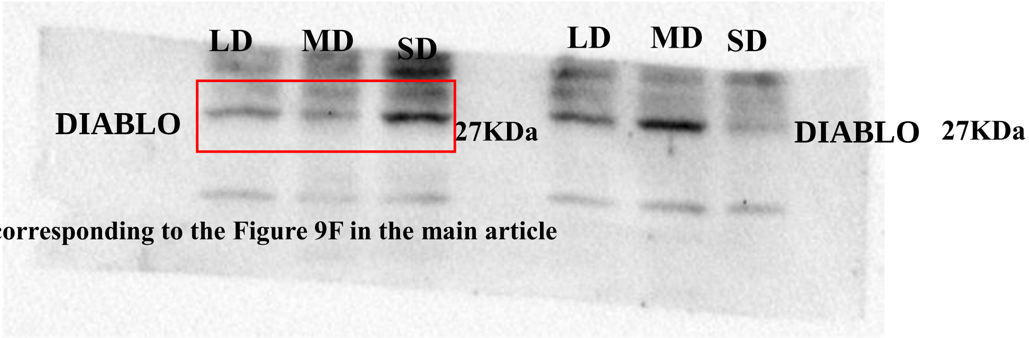

corresponding to the Figure 9F in the main article

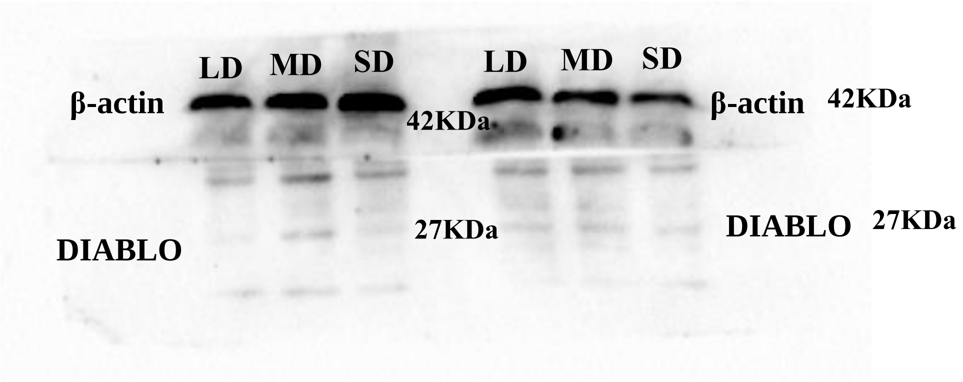

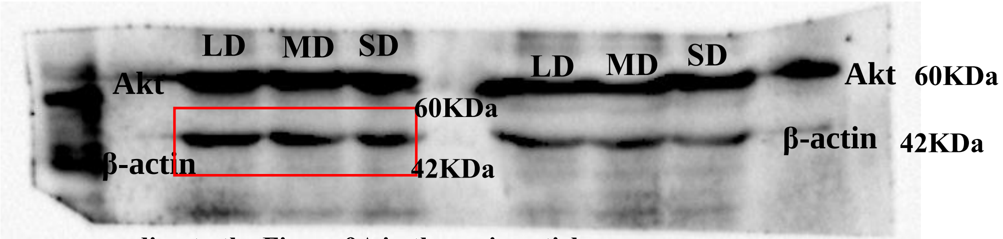

corresponding to the Figure 9A in the main article

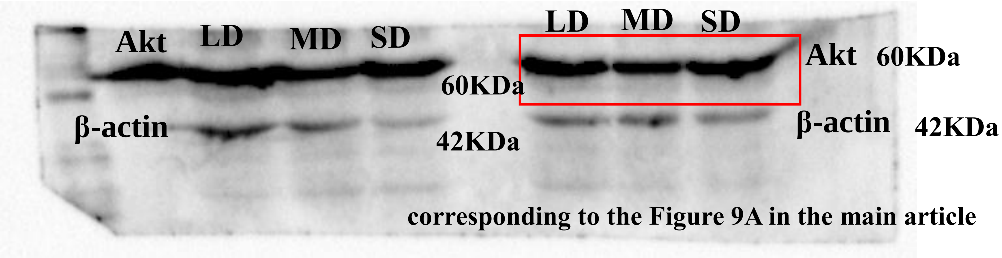
Akt
LD
MD
SD
60KDa
LD
MD
SD
Akt 60KDa
β-actin
42KDa
β-actin 42KDa
corresponding to the Figure 9A in the main article

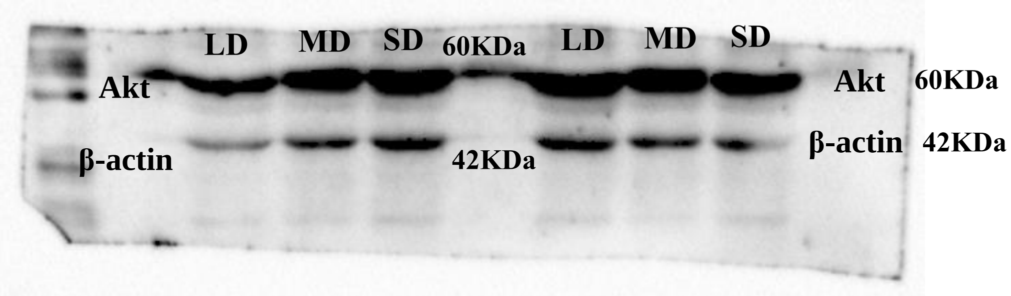
LD
MD
SD
60KDa
LD
MD
SD
Akt
Akt 60KDa
β-actin
42KDa
β-actin 42KDa

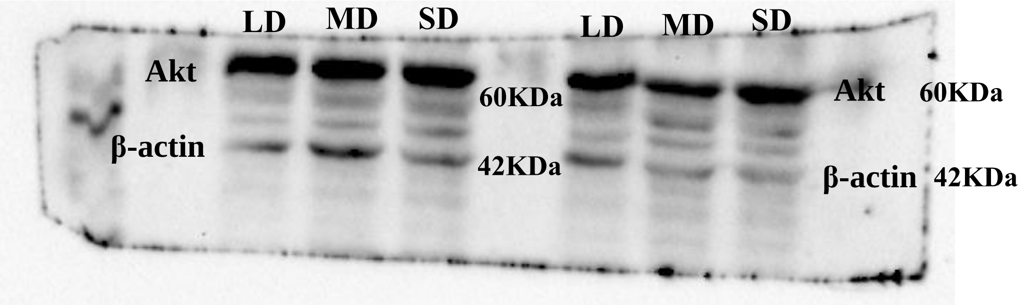
LD
MD
SD
LD
MD
SD
Akt
60KDa
Akt 60KDa
β-actin
42KDa
β-actin 42KDa

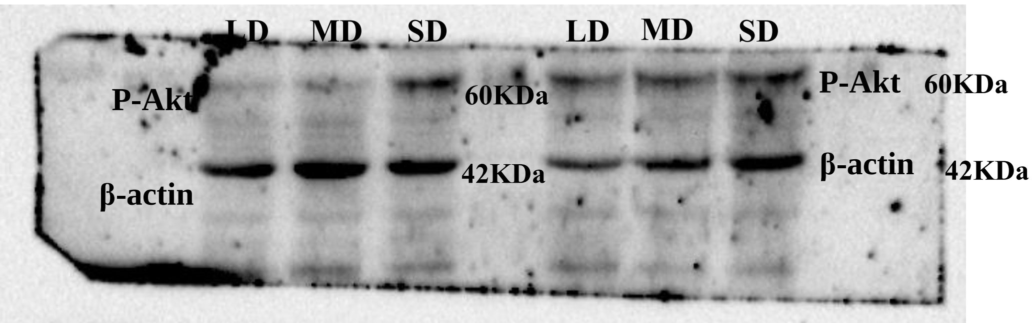
LD
MD
SD
LD
MD
SD
P-Akt
60KDa
P-Akt 60KDa
β-actin
42KDa
β-actin 42KDa

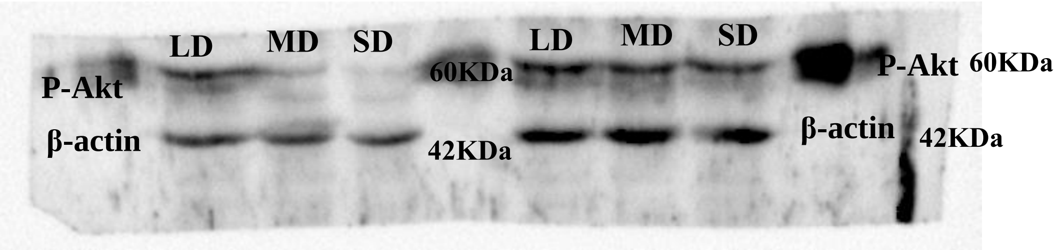
LD
MD
SD
LD
MD
SD
P-Akt
60KDa
P-Akt 60KDa
β-actin
42KDa
β-actin 42KDa

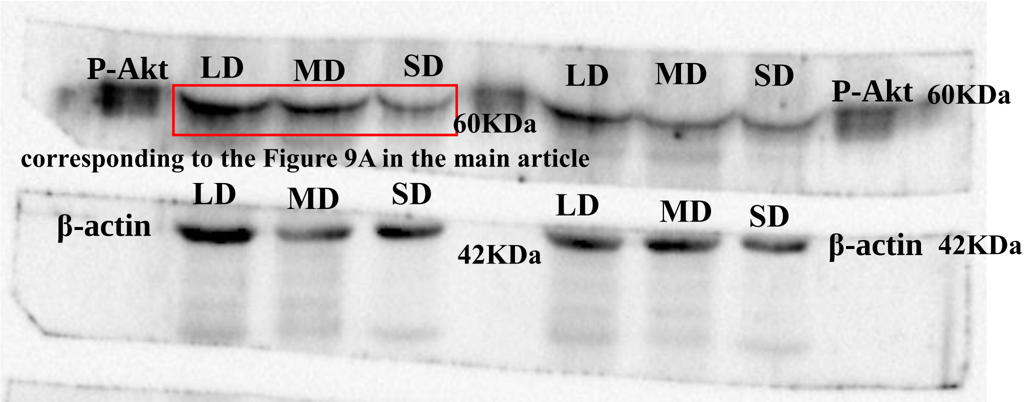
P-Akt
LD
MD
SD
60KDa
LD
MD
SD
P-Akt
60KDa
corresponding to the Figure 9A in the main article
β-actin
LD
MD
SD
42KDa
LD
MD
SD
β-actin
42KDa

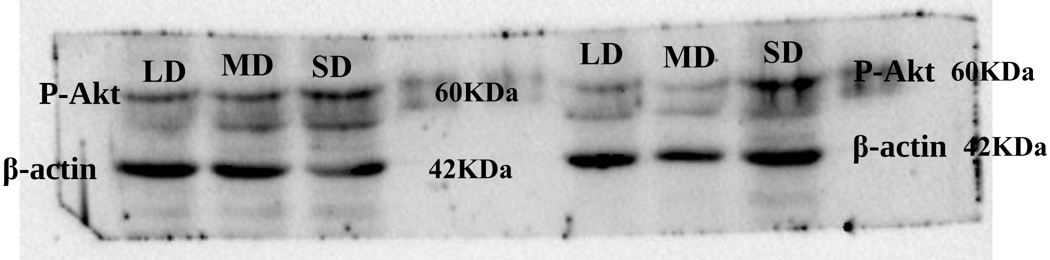
P-Akt
LD
MD
SD
60KDa
β-actin
42KDa
LD
MD
SD
P-Akt
60KDa
β-actin
42KDa
