# Supplementary material for: The Photoperiod Regulates Granulosa Cell Apoptosis through the FSH-Nodal/ALK7 Signaling Pathway in Phodopus sungorus
Source: Animals (Basel). 2022 Dec 16;12(24):3570. doi: 10.3390/ani12243570 (PMC9774567; doi:10.3390/ani12243570)
Supplement: Supplementary file 1 [file animals-12-03570-s001.zip › animals-2017514-supplementary-minor/Supplementary Files.pdf]

Supplementary Materials

Table S1. Detailed information of Immunofluorescence co-localization antibody.

Antibody	Dilutions	Catalog number	Biotechnology Co., Ltd.
rabbit anti-Nodal antibody	1:2000	bs-12243R	Bioss
HRP-labeled Goat Anti-Rabbit IgG	1:500	GB23303	Servicebio
ACVR1C rabbit polyclonal antibody	1:200	12610-1-AP	Proteintech
Alexa Fluor® 488 labeled goat anti-rabbit IgG	1:400	GB25303	Servicebio

Table S2. Fluorescence quantitative primers sequences and related information.

Primer	Primer sequences	Product Length (bp)	Annealing temperature (°C)
Nodal	F:5'-TGTAGGAGGGTCAAGTTCCAGGTG-3'<br>R:5'-CATCCACATTCCTCCACAAC-3'	88	60
ALK7	F:5'-GGAGATCGTTGGTACTCAAGGTAAGC-3'<br>R:5'-GTTTCACAGCCAGCCCTAAGTCAG-3'	119	60
XIAP	F:5'- GGTCAGAGCACAGGAGACACTTTC-3'<br>R:5'-CTGAACTCACACCAGACTCACTTCG-3'	87	60
Smad4	F:5'- TCTTCAGCACCACCCGCCTATG-3'<br>R:5'- GAACACCAATACTCAGGAGCAGGATG-3'	114	61
Akt	F: 5'-CGCTTCTTTGCCAACATCGTGTG-3'<br>R: 5'-CTCATCGAAATACCTGGTGTCAGTCTC-3'	105	61
DIABO	F:5'- CTCCACCTTTCTGTCCCAAACCAC-3'<br>R:5'- CTGCCACACTTCATCTTCCTCCTG-3'	148	61
HtrA2	F:5'- CTGGTCTTCGGCCTGGTGAT-3'<br>R:5'- ACTGCCAGCTGTGATTGGGT-3'	103	60
Bax	F:5'- TGCTACAGGGTTTCATCCA-3'<br>R:5'- GGCACTCGCTCAACTTCT-3'	111	54
Bcl2	F:5'- TGTGGATGACCGAGTACCTGAACC-3'<br>R:5'-AGACAGCCAAGAGAAATCAAACAGAGG-3'	125	61
Bcl-xL	F:5'- CTGTGTGGAAAGCGTAGACAAGGAG-3'<br>R:5'- AAGGCTCTAGGTGGTCATTCAGGTAG-3'	92	60
β-actin	F:5'- GAGACCTTCAACACCCCAGC-3'<br>R:5'- ATGTCACGCACGATTTCCC-3'	263	59

Table S3. Detailed information of western blotting antibody.

Antibody	Dilutions	Catalog number	Biotechnology Co., Ltd.
Nodal rabbit polyclonal antibody	1:1000	DF7791	Affinity Biosciences
ACVR1C rabbit polyclonal antibody	1:1000	12610-1-AP	Proteintech
Smad4 rabbit polyclonal antibody	1:1000	10231-1-AP	Proteintech
XIAP rabbit antibody	1:1000	AF6368	Affinity Biosciences
Akt rabbit Polyclonal antibody	1:1000	10176-2-AP	Proteintech
DIABLO rabbit polyclonal antibody	1:1000	10434-1-AP	Proteintech
HtrA2 rabbit polyclonal antibody	1:1000	15775-1-AP	Proteintech
Bax rabbit polyclonal antibody	1:5000	50599-1-AP	Proteintech
Bcl2 rabbit Polyclonal antibody	1:1000	26593-1-AP	Proteintech
Bcl-xL rabbit polyclonal antibody	1:1000	10783-1-AP	Proteintech
Beta actin rabbit polyclonal antibody	1:2000	20536-1-AP	Proteintech
GAPDH rabbit polyclonal antibody	1:2000	10494-1-AP	Proteintech
Alpha Tubulin rabbit polyclonal antibody	1:2000	11224-1-AP	Proteintech
IRDye 800 CW goat anti-rabbit secondary antibody	1:5000	31460	Thermo Fisher Scientific

Table S4. Numbers of growing follicles in each section under different photoperiods with the same magnification.

Slice number	SD	MD	LD
1-1	1	2	8
1-2	2	1	2
1-3	2	2	3
2-1	1	3	6
2-2	1	1	6
2-3	3	1	1
3-1	0	3	7
3-2	2	3	5
3-3	2	2	4
4-1	4	3	4
4-2	3	3	2
4-3	4	2	2
5-1	3	5	6
5-2	3	3	4
5-3	3	3	4
6-1	1	1	7
6-2	0	3	8
6-3	0	2	5

Mean	1.945	2.389	4.667

Table S5. Numbers of antral follicles in each section under different photoperiods with the same magnification.

Slice number	SD	MD	LD
1-1	0	1	1
1-2	0	0	1
1-3	0	1	1
2-1	0	1	4
2-2	0	0	4
2-3	1	0	3
3-1	0	1	2
3-2	0	1	2
3-3	0	0	0
4-1	0	0	0
4-2	0	0	0
4-3	1	01	0
5-1	0	11	0
5-2	0	1	0
5-3	0	1	2
6-1	0	0	2
6-2	0	0	3

6-3	0	0	0
Mean	0.056	0.444	1.167

Table S6. Numbers of atretic follicles in each section under different photoperiods with the same magnification.

Slice number	SD	MD	LD
1-1	2	0	1
1-2	2	1	0
1-3	5	2	0
2-1	2	2	0
2-2	3	1	0
2-3	3	1	2
3-1	4	2	1
3-2	3	2	1
3-3	4	2	0
4-1	2	0	1
4-2	2	0	1
4-3	2	2	1
5-1	1	2	1
5-2	1	3	1
5-3	1	2	0
6-1	3	1	1

6-2	4	1	1
6-3	5	1	0
Mean	2.722	1.389	0.667
